# Phylotranscriptomics Illuminates the Placement of Whole Genome Duplications and Gene Retention in Ferns

**DOI:** 10.3389/fpls.2022.882441

**Published:** 2022-07-14

**Authors:** Jessie A. Pelosi, Emily H. Kim, W. Brad Barbazuk, Emily B. Sessa

**Affiliations:** ^1^Department of Biology, University of Florida, Gainesville, FL, United States; ^2^Department of Microbiology and Cell Science, University of Florida, Gainesville, FL, United States; ^3^Genetics Institute, University of Florida, Gainesville, FL, United States

**Keywords:** fern, transcriptome, phylogenetics, polyploidy, whole genome duplication, biased gene retention

## Abstract

Ferns are the second largest clade of vascular plants with over 10,000 species, yet the generation of genomic resources for the group has lagged behind other major clades of plants. Transcriptomic data have proven to be a powerful tool to assess phylogenetic relationships, using thousands of markers that are largely conserved across the genome, and without the need to sequence entire genomes. We assembled the largest nuclear phylogenetic dataset for ferns to date, including 2884 single-copy nuclear loci from 247 transcriptomes (242 ferns, five outgroups), and investigated phylogenetic relationships across the fern tree, the placement of whole genome duplications (WGDs), and gene retention patterns following WGDs. We generated a well-supported phylogeny of ferns and identified several regions of the fern phylogeny that demonstrate high levels of gene tree–species tree conflict, which largely correspond to areas of the phylogeny that have been difficult to resolve. Using a combination of approaches, we identified 27 WGDs across the phylogeny, including 18 large-scale events (involving more than one sampled taxon) and nine small-scale events (involving only one sampled taxon). Most inferred WGDs occur within single lineages (e.g., orders, families) rather than on the backbone of the phylogeny, although two inferred events are shared by leptosporangiate ferns (excluding Osmundales) and Polypodiales (excluding Lindsaeineae and Saccolomatineae), clades which correspond to the majority of fern diversity. We further examined how retained duplicates following WGDs compared across independent events and found that functions of retained genes were largely convergent, with processes involved in binding, responses to stimuli, and certain organelles over-represented in paralogs while processes involved in transport, organelles derived from endosymbiotic events, and signaling were under-represented. To date, our study is the most comprehensive investigation of the nuclear fern phylogeny, though several avenues for future research remain unexplored.

## Introduction

**FIGURE 1:**
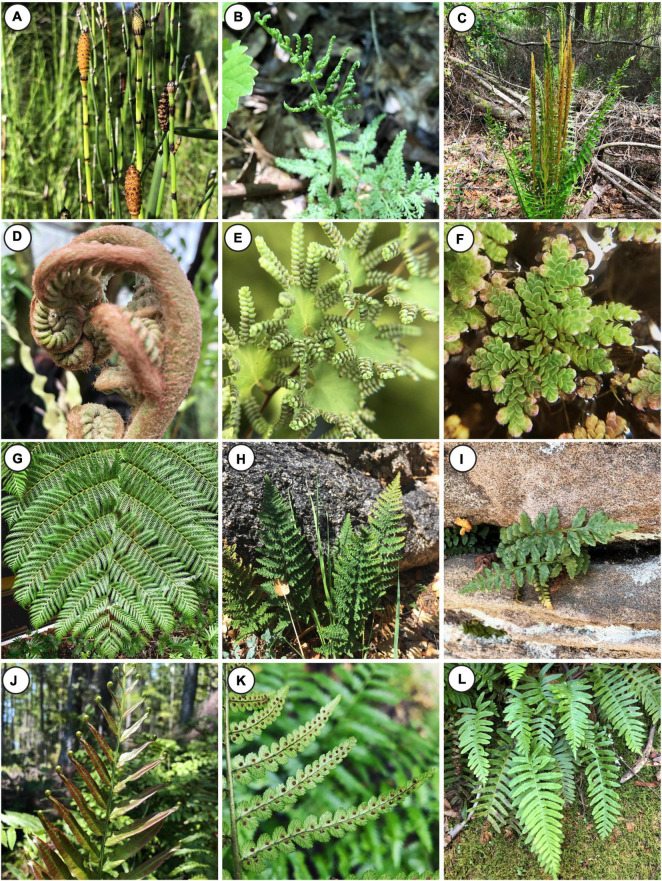
The diversity of ferns. **(A)** *Equisetum hyemale* (Equisetaceae, Equisetales), **(B)** *Sceptridium dissectum* forma *dissectum* (Ophioglossaceae, Ophioglossales), **(C)** *Osmundastrum cinnamomeum* (Osmundaceae, Osmundales), **(D)** *Angiopteris evecta* (Marattiaceae, Marattiales), **(E)** *Lygodium microphyllum* (Lygodiaceae, Schizaeales), **(F)** *Azolla filiculoides* (Salviniaceae, Salviniales), **(G)** *Sphaeropteris cooperi* (Cyatheaceae, Cyatheales), **(H)** *Myriopteris wootonii* (Pteridaceae, Polypodiales), **(I)** *Asplenium* hybrid (Aspleniaceae, Polypodiales), **(J)** *Telmatoblechnum serrulatum* (Blechnaceae, Polypodiales), **(K)** *Dryopteris ludoviciana* (Dryopteridaceae, Polypodiales), **(L)** *Polypodium virginianum* (Polypodiaceae, Polypodiales). All images by JP.

Ferns are the second largest group of vascular plants (with around 10,000 species, The Pteridophyte Phylogeny Group, 2016), the sister group to seed plants, and highly diverse (Figure 1). Molecular data have revolutionized our understanding of fern phylogenetics over the last two decades. Results from these studies have clarified the phylogenetic placement of ferns in the land plant phylogeny as sister to seed plants (Pryer et al., 2001; Wickett et al., 2014; One Thousand Plant Transcriptomes Initiative, 2019; henceforth, 1KP), the placement of Equisetales (horsetails) and Psilotales (whisk ferns) as true ferns (Pryer et al., 2001; Knie et al., 2015), the recent origins of most extant fern diversity (Schneider et al., 2004; Schuettpelz and Pryer, 2009; Testo and Sundue, 2016), deep (Pryer et al., 2001, 2004; Schuettpelz and Pryer, 2007; Kuo et al., 2011, 2018a; Rothfels et al., 2015; Testo and Sundue, 2016) and shallow (e.g., Rothfels et al., 2008; Sessa et al., 2012a; Schuettpelz et al., 2016; Xu et al., 2019; Kinosian et al., 2020) relationships of many fern lineages, and the role of polyploidy in shaping fern evolution (Manton, 1950; Klekowski and Baker, 1966; Sessa et al., 2012b; Schneider et al., 2017). Despite this progress, however, several key divergences along the backbone of the fern phylogeny remain unresolved, including the relationships between the eusporangiate and leptosporangiate ferns (Pryer et al., 2001; Knie et al., 2015; One Thousand Plant Transcriptomes Initiative, 2019), Gleicheniales and Hymenophyllales (Rothfels et al., 2015; Kuo et al., 2018a), among the sister groups to the eupolypods (Rothfels et al., 2015; Testo and Sundue, 2016), and within the eupolypods II (Aspleniineae *sensu* Rothfels et al., 2012; Testo and Sundue, 2016; The Pteridophyte Phylogeny Group, 2016; Wei et al., 2017; Du et al., 2021). Many challenges make resolving these deep relationships difficult, including lineage-specific rate heterogeneity, nuclear-plastid incongruence, and polyploidy.

The vast majority of DNA-based studies of fern phylogeny and evolution to date have used primarily or exclusively plastid loci (e.g., Pryer et al., 2001, 2004; Schuettpelz and Pryer, 2007; Testo and Sundue, 2016; see Table 1 in Rothfels et al., 2015 for a summary of the main studies in deep fern phylogenetics), which act as a single linkage group (Lynch, 2007) and are maternally inherited in ferns (Gastony and Yatskievych, 1992; Vogel et al., 1998; Guillon and Raquin, 2000; Kuo et al., 2018b). Nuclear analyses of fern phylogenetics, in contrast, have lagged behind and have focused on just a few loci. Large-scale comparative studies at the genomic scale are also lacking within ferns (Marks et al., 2021; Szövényi et al., 2021), although several genome-sequencing projects have been recently completed (Li F. W. et al., 2018; Marchant et al., 2019; Huang et al., 2022) or are in progress (Cheng et al., 2018; Pelosi unpub. data). The average homosporous fern (including 99% of fern species, Haufler et al., 2016; The Pteridophyte Phylogeny Group, 2016) has a 1C genome size of 12.05 pg (Sessa and Der, 2016), and there is a staggering 282-fold difference in genome sizes across all ferns (both homosporous and heterosporous), from 0.26 Gb in the heterosporous water fern *Salvinia cucullata* to 73.19 Gb in *Tmesipteris elongata* (Hidalgo et al., 2017; Li F. W. et al., 2018, respectively) (note that heterosporous ferns, which make up ∼1% of fern diversity, have substantially smaller genomes, with an average 1C value of 2.43 pg, Sessa and Der, 2016). For these reasons, whole genome studies across the fern clade are generally unfeasible with current sequencing and assembly technology. The implementation of transcriptome sequencing for phylogenetic study has been applied throughout the green plants (e.g., Wickett et al., 2014; One Thousand Plant Transcriptomes Initiative, 2019) and within ferns specifically in a small number of studies (Qi et al., 2018; Shen et al., 2018); these datasets, however, have not yet been combined and thoroughly interrogated. The enormous genomes of ferns, which may be a consequence of multiple rounds of whole genome duplication (WGD), and consequent difficulty with phasing alleles, parsing homeologs, and chimeric assemblies, have hampered the progress of nuclear-based phylogenetic studies in ferns.

Whole genome duplication, or polyploidy, is associated with nearly one-third of speciation events in ferns (Wood et al., 2009). Shifts in ecological niches (Marchant et al., 2016), phenotypes (Finigan et al., 2012), and environmental robustness (Van de Peer et al., 2017), or genetic changes such as biased gene retention (Van de Peer et al., 2017; Li Z. et al., 2018) and expression (Shan et al., 2020), and alternative splicing patterns (Zhou et al., 2011) may arise following WGDs. Genomes may also undergo large-scale post-polyploidy reorganizations (reviewed by Soltis et al., 2015); for example, following an allopolyploidy event (hybridization of two species accompanied by genome duplication), one subgenome often becomes “dominant” over the other (Alger and Edger, 2020; but see Krabbenhoft et al., 2021 for an example of extreme subgenome stability following an ancient duplication event). There are also several potential fates of individual duplicated genes (reviewed in Li et al., 2021). In most cases, one of the duplicate copies becomes non-functional, and will either be retained in the genome as a pseudogene or lost in the process of reorganization (for examples of gene silencing in *Tragopogon* see Buggs et al., 2011, 2010a,b). Alternatively, one of the duplicates may undergo neofunctionalization, where less effective purifying selection on one duplicate allows it to evolve a new function. A third possibility, sub-functionalization, posits that both duplicates retain complementary functions of the single pre-duplication gene. Alternatively, according to the Dosage Balance Hypothesis (DBH, Papp et al., 2003), genes with multiple interaction partners (such as transcription factors) are preferentially retained in duplicate following a WGD (Freeling, 2009; Defoort et al., 2019), implying that duplicates from small-scale events and WGDs should have different functions than those retained from polyploidy events. Several studies (e.g., Barker et al., 2008; Li et al., 2016; Li Z. et al., 2018) have found that duplicates from WGDs were enriched for different functions than the entire transcriptome or genome, and that these retained duplicates tended to converge on similar functions. Similar patterns and processes of genome evolution have yet to be explored in ferns.

There have been, on average, between two and four duplication events inferred in the ancestry of each extant fern species (One Thousand Plant Transcriptomes Initiative, 2019), with 19 (Huang et al., 2019) to 21 (One Thousand Plant Transcriptomes Initiative, 2019) putative events across the fern phylogeny. A comprehensive understanding of the evolution and biology of ferns requires thorough study of the placement and aftermath of WGD events throughout the history of this clade. Here, we leverage publicly available data to reconstruct nuclear phylogenies for ferns. We use this phylogenetic background to investigate (1) the phylogenetic backbone of ferns, (2) the placement of WGDs throughout the fern phylogeny, and (3) the fates and nature of duplicated genes following WGDs.

## Materials and Methods

### Transcriptome Assembly and Annotation

Sequence data from Qi et al. (2018) and Shen et al. (2018) were downloaded from the NCBI SRA. The quality of raw sequences was assessed with FastQC^1^ and reads were trimmed of adaptors and the first five bp with Trimmomatic ver. 0.39 (Bolger et al., 2014). Transcriptomes were assembled following the methods in One Thousand Plant Transcriptomes Initiative (2019); trimmed reads were assembled with SOAPdenovoTrans ver. 1.03 (Xie et al., 2014) with a kmer size of 25 bp. To remove any plastid sequence data that may have ended up in the nuclear assemblies, available fern plastome sequences were downloaded from NCBI (accessed July, 2020). BLASTN (Altschul et al., 1990) was used to compare assemblies against the plastome database and scaffolds or contigs with significant hits (*e*-value < 1 × 10^–4^, overlap > 300 bp, bitscore > 50) were removed from the assembly. Sequences with greater than or equal to 98% similarity were clustered with CD-HIT ver. 4.6.8 (Fu et al., 2012) to reduce transcript redundancy. Assemblies from 1KP were downloaded from the Cyverse repository for downstream analyses. Transcriptome completeness was assessed using BUSCO ver. 3.02 (Simão et al., 2015) with eukaryota odb9 lineage database (see Supplementary Appendix 1). Peptide and coding sequence (CDS) files were generated for each transcriptome using TransDecoder ver. 5.5.0 (Grabherr et al., 2011). We used Kruskal–Wallis tests by rank to determine whether assemblies from different studies were significantly different from one another for several metrics of interest, followed by pairwise comparisons using Wilcoxon rank sum tests with corrections for multiple testing using the Holm method (Holm, 1979).

### Phylotranscriptomics

Peptide and corresponding CDS files for the outgroups *Amborella trichopoda, Arabidopsis thaliana, Physcomitrella patens*, and *Selaginella moellendorffii* were downloaded from Ensembl Plants 51 (Howe et al., 2020). To ensure that all major lineages of land plants were represented, peptide and CDS files for the gymnosperm *Ginkgo biloba* were also downloaded from Guan et al. (2019). Peptide files from outgroups and ferns with >55% BUSCO completeness (92.6%, 239 of 258) were passed to OrthoFinder ver. 2.3.11 (Emms and Kelly, 2015, 2019) to identify orthogroups (OGs). OGs identified with OrthoFinder were filtered using the custom bash script *get_orthogroups.sh* to generate datasets for single- and multi-copy datasets. OGs that were single-copy and contained at least 60, 75, and 85% of the input transcriptomes were used to generate the “SCO60,” “SCO75,” and “SCO85” datasets. Multi-copy OGs that contained all transcriptomes were used to generate the “MCO” dataset.

The custom python script *extract_cds.py* (modified from Kasey K. Pham, pers. comm.) was used to obtain the corresponding coding sequences for each taxon in the filtered OGs. Sequences were aligned with the codon-aware alignment program MACSE ver. 2.0.4 (Ranwez et al., 2011) and gappy sites were removed with trimAl ver. 1.2 (Capella-Gutiérrez et al., 2009) by retaining sites that contained at least 50% of tips. Maximum likelihood gene trees were constructed from both the nucleotide and peptide alignments with IQTREE2 ver. 2.1.2 (Minh et al., 2020), with ModelFinder (Kalyaanamoorthy et al., 2017) and 1000 ultrafast bootstraps (Hoang et al., 2018) on 2 CPU threads where possible based on the recommendations from Shen et al. (2020). Note that trees constructed from the concatenated matrices required additional RAM and could not be run on only two CPU threads; these deviations are specified in our code. The optimal maximum likelihood gene trees for each locus were passed to Astral ver. 5.7.7 (Zhang et al., 2018) for the SCO datasets and Astral Pro ver. 1.1.2 (Zhang et al., 2020) for the MCO dataset to generate species trees under the multispecies coalescent (MSC). Concatenated alignments were used to generate a maximum likelihood species tree for the SCO datasets following the methods above, partitioned by locus (Chernomor et al., 2016). Discordance in the data was visualized using DiscoVista (Sayyari et al., 2018) and generalized Robinson-Foulds (GRF) distances between estimated species trees were calculated using the R package phangorn ver. 2.5.5 (Schliep, 2011).

Given heterogeneity among lineages (see Section “Discussion”), we compared the results from traditional models of sequence evolution to GHOST models (Crotty et al., 2020). GHOST is a mixture-model that takes a user-supplied number of components (*k*) and was developed to account for heterotachous evolution in datasets. For each locus in the SCO60 NT dataset, we ran IQTREE2 using the best-fitting model with classes *k* = 2–6 in both the linked and unlinked implementations of GHOST. The best number of classes was assessed using AIC. The optimal trees for the best-fitting number of classes under the GHOST model were passed to ASTRAL ver. 5.7.7 (Zhang et al., 2018) to construct the species tree as above. We also used a custom python script (*extract_codons.py*) written with the BioPython ver.1.79 module (Cock et al., 2009) to extract the first and second codon positions and third codon position from each untrimmed locus alignment in the SCO60 NT dataset. We then used trimAl on the first and second and third codon position alignments to remove sites with less than 50% tip occupancy. Gene trees were generated as above with IQTREE2 which were used to construct a species tree with ASTRAL.

Given that the phylogenetic position of two samples (*Onoclea sensibilis* ONSE and *Plagiogyria japonica* PLJA) suggested misidentifications, we extracted the longest plastid contig or scaffold from the initial assembly and used BLASTN (Altschul et al., 1990) against the nr database to determine their appropriate identification. The *Onoclea sensiblis* ONSE sample matched the *Matteuccia struthiopteris* chloroplast genome (KY427353.1) with a 98.7% identity compared to 91.26% identity to the *O. sensibilis* chloroplast genome (KY427354.1). The *P. japonica* PLJA sample matched the *P. subadnata* chloroplast genome (MN623362.1) with 99.25% identity. These samples have been named according to their respective hits for downstream analyses.

In a supplemental analysis, we downloaded sequence data from Dong et al. (2019) for *Sphaeropteris lepifera* and assembled the transcriptome as above. Coding sequences of *Cystodium sorbifolium* and *Saccoloma campylurum* from Qi et al. (2018) were provided by the author as the raw sequence files were corrupted and not available on the NCBI SRA. For these three transcriptomes, we used BLASTP (Altschul et al., 1990) to identify significant hits to the 2884 OGs in the SCO60 dataset. The contig or scaffold with the highest bit-score was extracted and nucleotide alignments, gene trees, and species trees were constructed as above.

### Divergence Time Estimation

We used SortaDate (Smith et al., 2018) to calculate the root-to-tip variance and bipartition support for each locus in the SCO60 NT dataset relative to the SCO60 NT MSC species tree. We then selected loci that were above the 90th percentile for bipartition support and below the 15th percentile for root-to-tip variance. This filtered dataset contained 99 single copy loci and was 136,137 bp in length. We generated a maximum likelihood tree with IQTREE2 as above using the SCO60 NT MSC species tree as a topological constraint to generate branch lengths relative to the number of substitutions per site. The resulting tree was then used to generate a dated phylogeny under a penalized likelihood method with treePL (Smith and O’Meara, 2012) with fifteen fossils (Supplementary Appendix 2) as calibration points along the phylogeny. To account for uncertainty in the dataset, we generated 100 bootstrap alignments from the 99 locus matrix and constructed trees from these bootstrap alignments using RAxML ver. 8.2.12 (Stamatakis, 2014). We then ran treePL on each of the bootstrap trees with three cross-validation runs and summarized them with treeAnnotator in BEAST ver. 2.5.0 (Suchard et al., 2018). We used the same methodology to date the supplemental tree with the inclusion of *C. sorbifolium, S. campylurum*, and *S. lepifera* with two additional fossil calibrations: we placed *Cyathocaulis* fossils (Tidwell and Nishida, 1993; Lantz et al., 1999) at 113–145 Ma for the stem Cyatheaceae as in Schuettpelz and Pryer (2009) and Du et al. (2021) and we used a stem age of 100.5–113 Ma for Lindsaeaceae based on the Regaladgo et al. (2017) as in Du et al. (2021). Although we did attempt to use MCMCTree (Yang, 2007) to estimate divergence times, due to the size of the dataset (e.g., number of loci, number of tips), Bayesian dating analyses were unfeasible.

### Whole Genome Duplications

The program wgd ver. 1.1.1 (Zwaenepoel and Van de Peer, 2019) was used to generate paralog age distributions (K_*S*_ plots) for each transcriptome. Normal mixture models were fit to the K_*S*_ distributions using the R package mixtools ver. 1.2.0 (Benaglia et al., 2009); AIC values of models with one component were compared to those of models with more than one component to determine if fits were statistically different. The R package SiZer ver. 0.1–7 (Chaudhuri and Marron, 1999; Sonderegger, 2020) was used to detect significant changes in slope (at α = 0.05). Briefly, SiZer identifies changes in slope based on changes in the first derivative in the curve (Chaudhuri and Marron, 1999). With respect to K_*S*_ plots, changes, particularly increases, would represent deviations from the background paralog distribution. Uncorrected interspecific K_*S*_ values were calculated for select species using wgd’s one_v_one feature, which generates a K_*S*_ distribution of one-to-one orthologs for two taxa of interest. We compared the uncorrected K_*S*_ rates and subsequent WGD hypotheses with rates corrected using ksrates ver. 1.1.1 (Sensalari et al., 2022). Using a set of transcriptomes, ksrates accounts for differences in synonymous divergence attributable to lineage-specific rate heterogeneity using two focal taxa with an inferred WGD based on an initial K_*S*_ analysis and three to four outgroup taxa to correct K_*S*_ values. We selected taxa that represent major lineages and used BUSCO scores to inform our selection process.

Sets of transcriptomes were carefully selected based on hypotheses about the relative placement of WGDs from K_*S*_ plots and previous studies (Huang et al., 2019; One Thousand Plant Transcriptomes Initiative, 2019). Taxa were picked based on their phylogenetic position to represent major clades and we used transcriptome completeness (i.e., BUSCO scores) to aid in our decisions of which taxa to include. For taxa with multiple samples (e.g., *Psilotum nudum, Dicksonia antarctica*), we selected the assembly with the highest BUSCO score for our analyses. We extracted OGs where at least 45% of these transcriptomes were present at least once. OGs were aligned and gene trees were generated as above. Using subsets of the species tree and these gene trees, we performed several MAPS analyses (Li et al., 2015; Li Z. et al., 2018) to estimate the placement of WGDs in a phylogenomic context. Average gene birth (λ) and death (μ) rates for each dataset were estimated following Tiley et al. (2016). Briefly, we generated OGs using OrthoFinder for just the taxa of interest and filtered gene families to only include those with at least one copy in the outgroup and at least one copy in any other taxon in the dataset and removed any gene families with greater than 100 members in any taxon. We generated ten random subsets of 500 gene families to estimate λ and μ with WGDgc ver. 1.3 (Rabier et al., 2014) using the geometric mean of gene family size as the root prior and the “oneInBothClades” likelihood conditioning. To create a null distribution, we generated three sets of 1,000 simulated trees without a WGD using GuestTreeGen from PhyloGenData (Sjöstrand et al., 2013): 1,000 with the maximum likelihood values of λ and μ; 1,000 with λ and μ at half the ML values; and 1,000 with λ and μ at three times the ML values following (Li F. W. et al., 2018, Li Z. et al., 2018). We then generated 3,000 trees as above with WGDs in the midpoint of branches of interest (e.g., leading to nodes with higher subtree duplications relative to other nodes, nodes corresponding to K_*S*_ peaks) to create a positive distribution with a retention rate of 20%. We compared the MAPS analysis with the empirical data against these simulations using Fisher’s exact tests.

We further compiled haploid (gametophytic) chromosome counts (*n*) and 1C genome sizes from Fujiwara et al. (2021), the Chromosome Count Database (Rice et al., 2015), and the Kew Plant *C*-Values Database (Leitch et al., 2019). For more recent inferred WGDs and where there was a direct sister clade without an inferred WGD, we compared chromosome number and genome size of the inferred polyploid taxa to their sister clade.

### Gene Retention

Transcriptomes were blasted against the Araport11 *A. thaliana* protein dataset (Berardini et al., 2004). Gene ontology (GO) terms were sorted and tallied into GO Slim categories and visualized using custom perl scripts. Paralogs falling within ±1 SD of the mean of the putative WGD peak(s) and whose posterior probability was highest for the mixture component corresponding to the inferred WGD were also annotated using this pipeline. We used Chi-squared tests to determine if the GO composition of paralogs were significantly different from their respective full transcriptomes. Following Barker et al. (2008) and Shi et al. (2010), GO Slim categories with residuals >2 are considered to be overrepresented in paralog sequences compared to the full transcriptomes, while GO Slim categories with residuals <-2 are considered to be underrepresented. We further compiled GO annotations from all transcriptomes and paralogs for each WGD event identified along the phylogeny and compared the GO Slim composition of paralogs retained in each of these events.

## Results

### Assemblies

We compiled a total of 261 fern transcriptomes that represent 230 species; after filtering for transcriptome completeness, there were a total of 242 transcriptomes representing 211 species from 43 families (89.6% of 48 total families) in all 11 orders. We found that BUSCO completeness was significantly different between all three studies (Supplementary Figure 1A, χ^2^ = 74.565, *df* = 2, *P* < 0.001), as well as the total number of scaffolds and contigs (Supplementary Figure 1B, χ^2^ = 154.57, *df* = 2, *P* < 0.001). There were additional significant differences in total transcriptome length (Supplementary Figure 1C, χ^2^ = 75.388, *df* = 2, *P* < 0.001) and number of predicted genes (Supplementary Figure 1D, χ^2^ = 9.5959, *df* = 2, *P* < 0.01) in which the 1KP assemblies were significantly shorter and had fewer predicted genes compared to the assemblies from the Qi et al. (2018) and Shen et al. (2018) studies, but the other assemblies were not different from one another. Assembly statistics for transcriptomes from each of the studies are given in Table 1 and Supplementary Appendix 1. Note that these analyses and statistics do not include the assemblies for *C. sorbifolium* and *S. campylurum* since we only had access to the CDS files.

**TABLE 1:** Assembly statistics for transcriptomes from three publications used in this study.

Study	No. transcriptomes	No. contigs + scaffolds	Assembly length (Mbp)	No. predicted genes	% Complete BUSCO genes
Shen et al., 2018	69	**147425** (53777, 660237)	**55.2** (28.3, 132.8)	**26514** (17527, 47079)	**85.07** (48.51, 95.71)
Qi et al., 2018	119	**121550** (55147, 483673)	**53.6** (24.1, 111.7)	**26883** (15893, 46757)	**87.74** (50.83, 96.04)
One Thousand Plant Transcriptomes Initiative, 2019	70	**13241** (61, 21403)	**36.7** (9.9, 67.7)	**23095** (648, 39285)	**66.44** (0, 94.39)
Total	258	**99084** (61, 660237)	**49.5** (9.9, 132.8)	**25756** (648, 47079)	**81.25** (0, 96.04)

*Metric averages are given in bold, followed by minimum and maximum in parentheses: **average** (minimum, maximum).*

### Clustering and Phylogenetics

OrthoFinder identified a total of 6,507,715 genes from 244 transcriptomes (239 ferns and 5 outgroups). Nearly all genes (94.9%) were assigned to one of 126,747 shared OGs. The mean and median OG size was 48.7 and 3.0 genes, respectively, with 1796 OGs that were represented at least once per transcriptome. After filtering and trimming gappy sites (see Section “Materials and Methods”), 2884, 1161, and 135 single-copy OGs were retained in the SCO60, SCO75, and SCO85 datasets, respectively (Table 2). A total of 1585 multi-copy OGs were retained in the MCO dataset (Table 2).

**TABLE 2:** Datasets constructed in this study and corresponding metrics.

Type of data	Dataset name	Percent of transcriptomes present	Number of orthogroups after filtering	Median orthogroup length (bp/AA)	Mean orthogroup length (bp/AA)	Total length (bp/AA)
Multi-copy	MCO	100%	1585	1,326.0 462.3	1,390.0 441	2,203,082 732,267
Single-copy	SCO60	60%	2884	925.5	990.4	2,856,366
				307.5	329.1	949,237
	SCO75	75%	1161	768.0	795.7	923,894
				225.0	264.3	306,805
	SCO85	85%	135	690.0	712.3	96,165
				229.0	236.4	31,920

*AA, amino acid; bp, base pair.*

**FIGURE 2:**
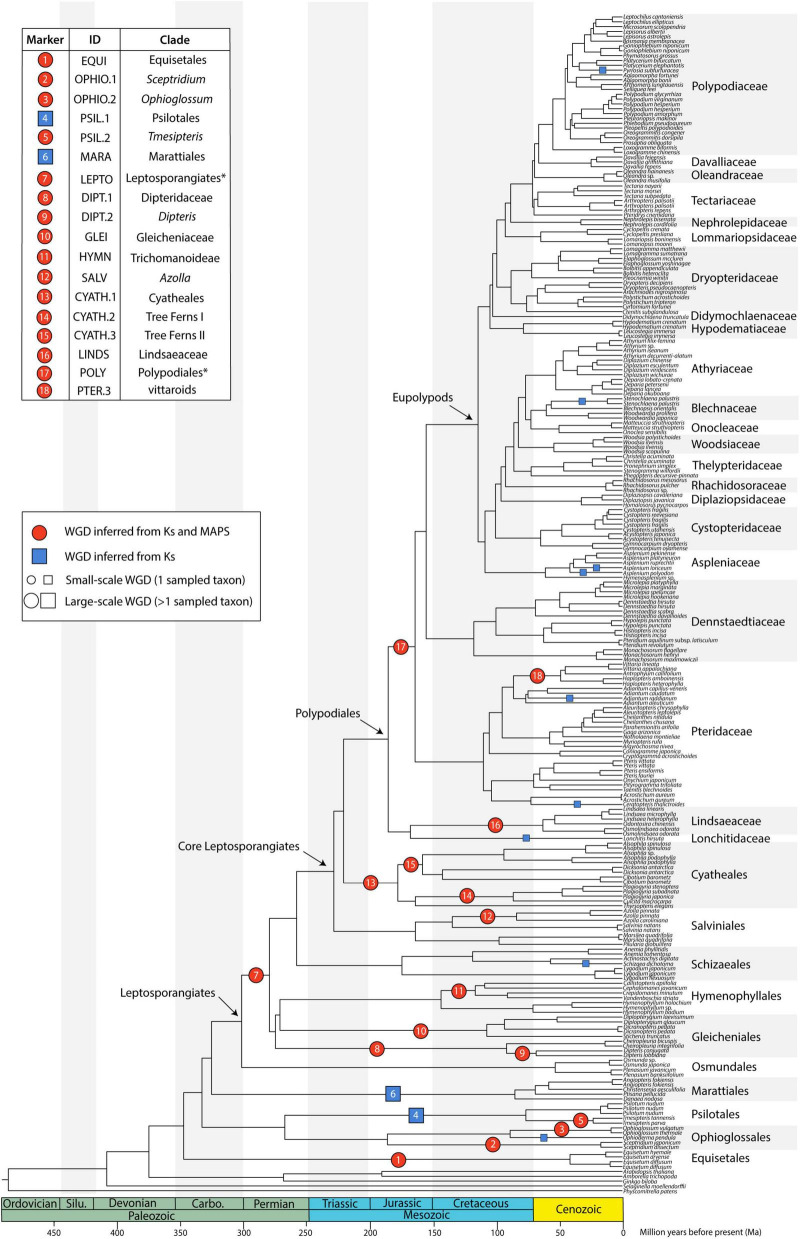
Species tree generated from 2884 single copy nuclear loci (SCO60 dataset) under the multi-species coalescent. Divergence times are based on a penalized likelihood method in TreePL. Inferred whole genome duplications (WGDs) are placed on the tree (note that the age of the WGDs are not depicted, rather events are placed on the midpoint of the branches). Events inferred from K_*S*_ and MAPS analyses are shown as red circles, and events inferred from K_*S*_ evidence only are shown as blue squares. The size of the symbol reflects whether the event is large-scale (larger symbol, including more than one sampled taxon) or small-scale (smaller symbol, including only one sampled taxon). Asterisks for LEPTO and POLY events indicate there are no current names for the clades corresponding to the taxa affected by these events: LEPTO is shared by leptosporangiate ferns excluding Osmundales and POLY is shared by Polypodiales excluding Lindsaeineae and Saccolomatineae.

**FIGURE 3:**
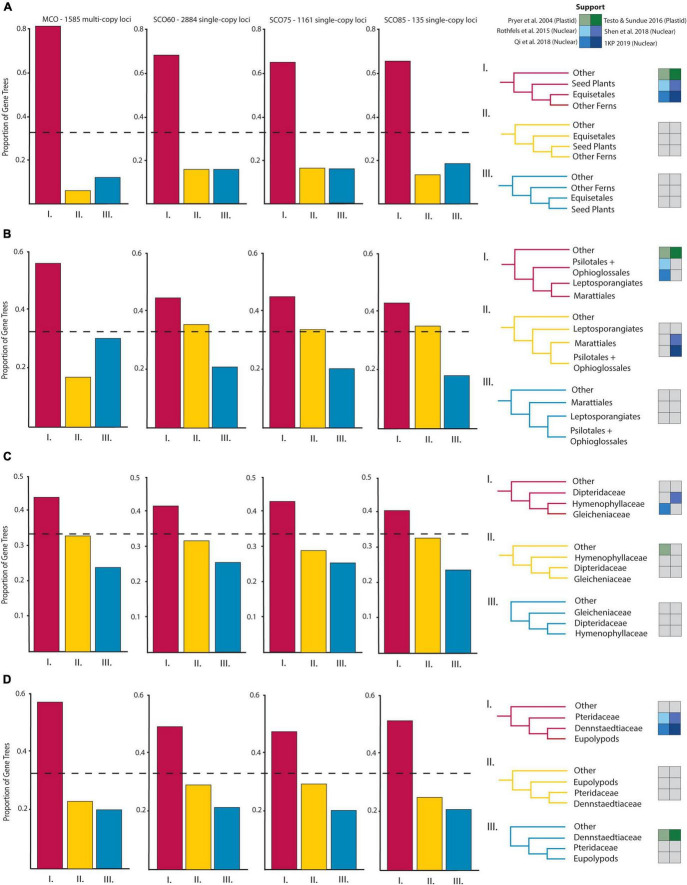
Proportions of gene trees supporting the inferred species trees and alternative topologies for relationships of particular interest: **(A)** Horsetails (Equisetales) and other ferns, **(B)** Eusporangiate-leptosporangiate fern relationships, **(C)** Gleicheniales and Hymenophyllales, and **(D)** Pteridaceae, Dennstaedtiaceae, and the eupolypods. Topological support from select plastid (green, Pryer et al., 2004; Testo and Sundue, 2016) and nuclear (blue, Rothfels et al., 2015; Qi et al., 2018; Shen et al., 2018; One Thousand Plant Transcriptomes Initiative, 2019) studies are given beside possible topologies.

Tree topology was generally consistent across analyses with most nodes having full or high support values (Figures 2, 3 and Supplementary Figure 2). All orders, except Gleicheniales, were monophyletic; all families were monophyletic, except for Tectariaceae and Athyriaceae in some analyses (see Section “Discussion”). In nearly all cases, genera that contained multiple samples were monophyletic, though some were not (e.g., *Cheilanthes*). GRF distances between trees were relatively low, with generalized scores all equal to or less than 0.06 (GRF ≤ 0.06; Supplementary Figure 3). Analyses were clustered first by the type of analysis [maximum likelihood on concatenated data matrix (ML) vs. multispecies coalescent (MSC) in ASTRAL], then generally by data type (nucleotide vs. amino acid), and finally by the dataset used (single copy, multi-copy, and percent of missing taxa). The maximum likelihood trees were the most similar to each other, having GRF ≤ 0.03, with MSC analyses differing by up to GRF = 0.06. There were clusters of low GRF values within the MSC analyses, where SCO75, SCO60, and MCO MSC analyses were clustered with GRF ≤ 0.02, with clusters for nucleotide and amino acid data types. The tree that differed the most from the other analyses was the SCO85 MSC analysis on amino acids, which had GRF values from 0.04 to 0.06 compared to other trees. Interestingly, the topology of both linked and unliked GHOST species trees were identical to the SCO60 NT MSC tree (GRF = 0, Supplementary Figures 2, 3). There were some topological differences when the first and second (CP12) and third (CP3) codon positions were analyzed separately in the SCO60 NT dataset. Tree topologies are compared in further detail for specific clades of interest in the Discussion.

Our penalized likelihood dating analysis resulted in family stem ages similar to those in previous studies (see Supplementary Appendix 3 and Supplementary Figures 4–6). We estimated that ferns originated around 346.7 Ma (range 342.5–346.7 Ma), leptosporangiate ferns around 300.9 Ma (range 299.3–312 Ma), Polypodiales around 185.0 Ma (range 172.2–222.0 Ma), and eupolypods around 114.0 Ma (range 79.2–123.6 Ma). With the three additional taxa and 17 fossil calibrations (see Section “Materials and Methods”) we estimated that ferns originated around 346.5 Ma (range 345.7–346.7 Ma), leptosporangiate ferns around 299.1 Ma (range 299–300.3 Ma), Polypodiales 166.9 Ma (range 160.6–181.9 Ma), and eupolypods around 95.3 Ma (range 86.2–111.6 Ma). Divergence times of specific families and clades are further compared in the Section “Discussion.”

### Whole Genome Duplications

**FIGURE 4:**
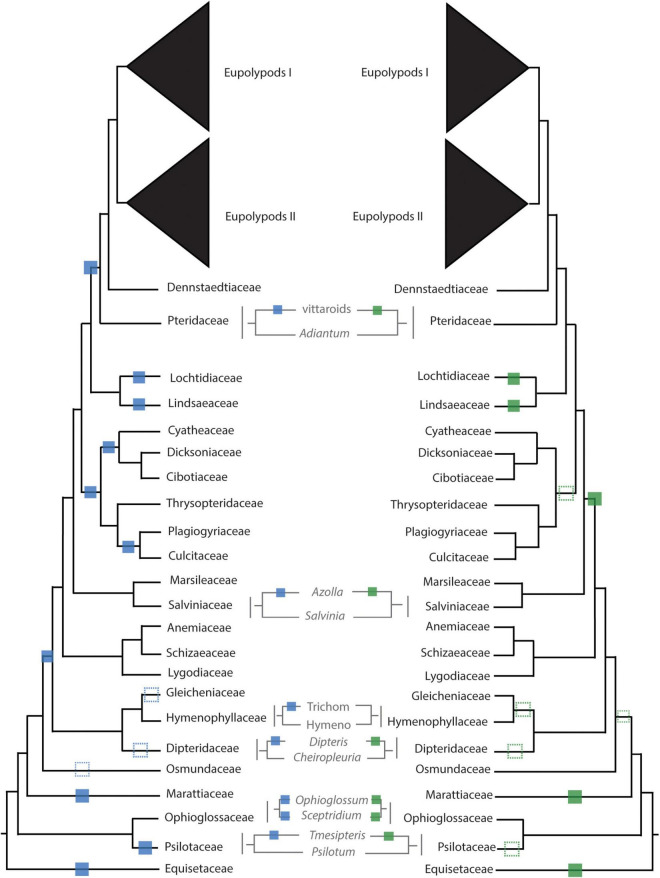
Inferred whole genome duplication (WGD) events from uncorrected (left, blue) and corrected (right, green) K_*S*_ analyses. Unambiguous placements of inferred WGDs are depicted as solid squares; ambiguous placements are depicted as dotted squares. Trichom = Trichomanoideae, Hymeno = Hymenophylloideae.

Of the 239 fern transcriptomes analyzed (not including the three additional taxa in the supplemental analyses), 193 had evidence of at least one peak in their K_*S*_ plots (80.75%); of these, 163 transcriptomes had one peak, and 30 had two peaks (Supplementary Appendix 4 and Supplementary Figure 7). Median peak K_*S*_ values ranged from 0.104 to 2.191, with nearly all inferred WGDs (25 out of 27) having median peak K_*S*_ < 2, suggesting that these analyses do not suffer from saturation effects. Briefly, at high synonymous substitution values (typically K_*S*_ > 2), a build-up of synonymous mutations not related to a duplication event may appear as a “saturation peak” in K_*S*_ plots (Vanneste et al., 2013), resulting in a false-positive inference of a WGD. The two events with median K_*S*_ > 2 were supported using MAPS (see below). In general, SiZer identified significant increases in slope consistent with K_*S*_ plots (Supplementary Figure 7), although there were several cases where significant increases were detected when no discernable K_*S*_ peaks were found (Supplementary Figure 7). Furthermore, SiZer failed to detect any significant increases in slope of K_*S*_ distributions of transcriptomes that had more than one peak, such as *Alsophila* spp. (Supplementary Figure 7). We conducted a total of 18 MAPS analyses that utilized 147,273 empirical gene trees with an average of 8663 gene trees per analysis (range 8043–11334), and 102,000 simulated gene trees (Supplementary Appendix 5). While WGD inferences from corrected and uncorrected K_*S*_ values were generally consistent, there were some instances where they conflicted (Figure 4). Using a combination of evidences, we inferred 27 large- and small-scale WGDs throughout the phylogeny (Figure 2, Supplementary Figures 8, 9, Supplementary Appendix 5); 18 large-scale WGDs (present in more than one sampled taxon) and 9 small-scale WGDs (present in only one sampled taxon). Sixteen of these WGDs were supported in both the MAPS and K_*S*_ analyses.

There were several independent, large-scale inferred WGD events in the extant eusporangiate ferns, with WGDs separately shared by Equisetales (EQUI), *Sceptridium* (OPHIO.1), *Ophioglossum* (OPHIO.2), Psilotales (PSIL.1), *Tmesipteris* (PSIL.2), and Marattiales (MARA) (Figure 2). A small-scale, unshared duplication in *Ophioderma pendula* (OPHIO.3) was also identified. In these lineages it was difficult to assess an additional lines of chromosome number or genome size evidence as there were no direct sister groups lacking an inferred WGD. For example, all *Equisetum* species sampled have *n* = 108 with a base chromosome number of *x* = 108, suggesting that they are all diploid (or ancient polyploids). Furthermore, repeated rounds of neopolyploidy or multiple cytotypes within a genus or species may obscure signals of paleopolyploidy events. For example, *Sceptridium, Ophioglossum*, and *Ophioderma* should have the same haploid chromosome number if they each underwent independent WGDs. However, the haploid chromosome number in *Sceptridium dissectum* is *n* = 45, while *Ophioglossum vulgatum* has *n* = 240–1320 and *O. pendula* has *n* = 370. In these instances, we could not use chromosome number as a line of evidence to support or reject our hypothesized WGD events. We do find chromosomal support for the PSIL.2 event, with the most common haploid chromosome number of *P. nudum* of *n* = 52 (although several counts have been reported ranging from *n* = 46–210), compared to *n* = 104 for *Tmesipteris tannensis*. Furthermore, the genome size estimated for *P. nudum* (1C = 32.8 pg) is just about half that reported for *T. tannensis* (1C = 74.84 pg), supporting our inference of the PSIL.2 WGD event.

While there was not a significant increase in duplicated gene trees relative to a null distribution in the putative WGD in Marattiales, there was a significant increase at the MRCA of *Angiopteris fokiensis* and *Ptisana pellucida* which is consistent with a WGD (Supplementary Appendix 5). However, both corrected and uncorrected K_*S*_ plots suggests a WGD shared by all Marattiales (Figure 4). Furthermore, the base chromosome numbers of genera in Marattiales do not show an increase at the node identified by the MAPS analysis alone (at the MRCA of *A. fokiensis* and *P. pellucida*). There are a range of haploid chromosome counts in Marattiales; *A. fokiensis* has *n* = 40, *Christensenia aesculifolia* has *n* = 80, and the base haploid chromosome number of *Ptisana* is *n* = 39, with no discernable pattern to confirm the within-Marattiales WGD event suggested by MAPS alone. While we cannot directly compare the karyotypes of Marattiales to a sister group that has not undergone a WGD, treating Osmundaceae as a diploid outgroup with *x* = 22 would further support the inference of a WGD at the base of Marattiales.

In the leptosporangiate ferns, we identified an event shared by all leptosporangiates excluding Osmundales (LEPTO, but see corrected K_*S*_ plots in Figure 4 and Supplementary Figure 9 for an alternative hypothesis). In the clade comprised of Hymenophyllales + Gleicheniales, we inferred four WGDs: events shared by Gleicheniaceae (GLEI), Dipteridaceae (DIPT.1), *Dipteris* (DIPT.2), and Trichomanoideae (HYMN). The placement of several of these events were ambiguous in both the corrected and uncorrected K_*S*_ analyses, while MAPS found strong support for the separate events. The DIPT.2 event requires further investigation, as the haploid chromosome number of *Dipteris conjugata* (*n* = 33) is nearly a quarter that of *Cheiropleuria dicuspis* (*n* = 116). Both *Dipteris* and *Cheiropleuria* have base chromosome numbers of *x* = 33, so it is possible the WGD event inferred here is a burst of gene duplications. HYMN is additionally supported by chromosome counts and genome sizes, although it is not identified in the corrected K_*S*_ analysis (Figure 4 and Supplementary Appendix 5). The sampled *Hymenophyllum* all have *n* = 21 or 22 and *H. badium* has a 1C genome size of 16.15 pg, values which are nearly half that of those taxa hypothesized to have undergone the HYMN WGD event in Tichomanoideae, which have *n* = 36 and 1C genomes sizes from 25.04 to 25.24 pg (*Crepidomanes minutum* and *Cephalomanes javanicum*, respectively). If we compare the chromosome numbers of taxa that underwent the proposed DIPT.1 and GLEI WGD events to *Hymenophyllum* species which do not have a lineage-specific WGD event, the DIPT.1 and GLEI taxa have greater haploid chromosome numbers (*n* = 33–116 in Dipteridaceae and *n* = 39 in Gleicheniaceae) compared to *Hymenophyllum* (*n* = 22). These shifts in chromosome number may coincide with the inferred WGDs. In Schizaeales, a single unshared event was inferred in *Schizaea dichotoma* (SCHIZ).

In Salvinales, we found evidence for the WGD identified by Li F. W. et al. (2018) and One Thousand Plant Transcriptomes Initiative (2019), shared by *Azolla* species (SALV). There is greater than double the number of chromosomes in a haploid *Azolla pinnata* genome (*n* = 22) compared to *Salvinia natans* (*n* = 9). Interestingly, *S. natans* has a much greater genome size (1C = 1.82 pg) compared to *Azolla filiculoides* (syn. *A.* cf *caroliniana*), though this is not the case in other *Salvinia* species (e.g., *S. culcutta* has 1C = 0.25 pg). Three major WGD events were inferred in Cyatheales, one at the base shared by the order (CYATH.1), one shared by Culcitaceae and Plagiogyriaceae (CYATH.2), and one shared by Cibotiaceae, Cyatheaceae, and Dicksoniaceae (CYATH.3). In the MAPS analysis of CYATH.2, the empirical proportion of gene trees duplicated at the corresponding node of interest was significantly greater than the null distribution, but less than the positive distribution (Supplementary Appendix 5). While there are clear peaks in uncorrected K_*S*_ plots and evidence from MAPS, chromosome numbers are relatively uniform in Cyatheales. For example, the taxa that are proposed to have undergone the CYATH.3 WGD event have haploid chromosome numbers *n* = 65–69, while *Thrysopteris elegans* has *n* = 78. Furthermore, genome sizes of *Cibotium barometz* and *Alsophila spinulosa* (1C = 4.48 and 7.36 pg, respectively) are lower than that of *T. elegans* (1C = 10.23 pg). A similar pattern is observed in the CYATH.2, though there is greater variation in chromosome number *n* = 68–130 in *Plagiogyria* and *Culcita*. When we evaluated corrected K_*S*_ values, we found that the most recent peaks thought to represent CYATH.2 and CYATH.3 were found to be older than the inferred position by MAPS. The corrected K_*S*_ analysis instead suggests a single duplication at the base of Cyatheales, with an older event shared by Polypodiales + Salviniales + Cyatheales. It is possible that these inferred events within Cyatheales are bursts of gene duplication; genome sequencing projects focused on the tree ferns are in progress (or have been recently published, Huang et al., 2022) and should aim to tackle this question. We inferred two independent events in Lindsaeineae, one event in *Lonchitis hirsuta* (LONCH) and one shared by Lindsaeaceae (LINDS).

In Polypodiales, there was one inferred WGD shared by the order excluding Lindsaeineae and Saccolomatineae (POLY). One shared event was identified in the vittaroids within Pteridaceae (PTER.3), along with two independent events in *Adiantum raddianum* (PTER.2) and *Ceratopteris thalictroides* (PTER.1). Relative to *Adiantum*, the vittaroid ferns had much higher haploid chromosome numbers (*n* = 120) and genome sizes (1C = 32.81 and 33.17 pg for *Antrophym callifolium* and *Vittaria lineata*, respectively), which are nearly quadruple the chromosome numbers (*n* = 29, 30) and six times greater the genome sizes (1C = 5.17–5.58 pg for *A. caudatum* and *A. aleuticum*) of diploid *Adiantum.* Within the eupolypods, several small-scale events were also identified in *Asplenium loriceum* (ASPL.2), *Asplenium polyodon* (ASPL.1), *Stenochlaena palustris* (BLECH), and *Pyrrosia subfuracea* (PYRO). Compared to the base chromosome numbers in their respective genera (*Asplenium x* = 36, *Stenochlaena* and *Pyrrosia x* = 37), these taxa have twice the number of haploid chromosomes except for *Pyrrosia subfurfuracea* (*n* = 37).

### Gene Retention Analyses

By comparing the number of putative paralogs within ±1SD of the K_*S*_ peak mean to the number of predicted genes from TransDecoder, we estimated that gene retention in ferns is low, with 11.97% of genes remaining in duplicate following an inferred WGD event (range 4.10–20.35%). Over- and under-represented GO Slim categories were similar throughout taxa and events (see Section “Discussion,” Supplementary Appendix 6, Supplementary Figures 11, 12) although lineage-specific differences are present. Generally, processes involved in binding (protein binding, nucleic acid binding, DNA binding, RNA binding), responses to stimuli (response to endogenous stimulus, response to abiotic stimulus, response to chemical), and certain organelles (nucleus, ribosome, endoplasmic reticulum, Golgi apparatus) were over-represented in paralogs. Processes involved in transport (transporter activity, transport, nuclear envelope), organelles derived from endosymbiotic events (mitochondrion, chloroplast), and signaling (signaling receptor binding and activity) were under-represented in paralogs.

## Discussion

### Resolving the Fern Phylogeny Backbone

Our understanding of the fern phylogeny has improved substantially over the last several decades, as our field has transitioned from morphological to molecular to genomic-based phylogenetic methods. The phylogenies we reconstructed here are largely consistent with the topologies from most recent plastid and nuclear analyses of ferns. Below we discuss several areas of the fern phylogeny that have been and remain difficult to resolve; for each clade we discuss relationships, gene tree-species tree conflict, and inferred ages, focusing primarily on the SCO60 NT MSC tree/dataset which contains the largest number of loci, and highlight points of discordance between this, our other datasets, and the literature.

#### Eusporangiate Ferns

The eusporangiate fern lineages form a successive grade to the leptosporangiate fern clade (Figure 2), with *Equisetum* sister to the rest of ferns, followed by a clade comprised of Ophioglossales and Psilotales, and Marattiales sister to the leptosporangiate ferns. We consistently found *Equisetum* to be sister to the rest of ferns (Figure 2 and Supplementary Figure 2) with relatively low conflict among gene trees (Figure 3A), which agrees with most recent findings (Kuo et al., 2011; Knie et al., 2015; Rothfels et al., 2015; Testo and Sundue, 2016; Qi et al., 2018; Shen et al., 2018; One Thousand Plant Transcriptomes Initiative, 2019), although some older studies have suggested *Equisetum* as sister to Marattiales (Pryer et al., 2001, 2004). Most previous studies have recovered a Devonian or Carboniferous origin of ferns, which is consistent with our finding a stem age of Equisetales of 346 Ma (range 341.5–347.0 Ma), although the age range in the literature spans 100 MY (Supplementary Figures 4–6), from 431 Ma (plastid data, Testo and Sundue, 2016) to 321 Ma (nuclear data, Shen et al., 2018). The placement of the clade consisting of Ophioglossales and Psilotales has differed across previous studies, with some suggesting that Ophioglossales and Psilotales together are sister to the rest of the ferns (Pryer et al., 2001, 2004), while most find them forming a grade leading up to leptosporangiate ferns (Knie et al., 2015; Rothfels et al., 2015; Testo and Sundue, 2016; Qi et al., 2018; Shen et al., 2018; One Thousand Plant Transcriptomes Initiative, 2019; Figure 2). The placement of Psilotales and Ophioglossales relative to each other and to other ferns was congruent among analyses (Supplementary Figure 2) and loci. A similar range of ages exists for the stem of Psilotales and Ophioglossales (Supplementary Figures 4–6), which we recovered as 267 Ma (range 226.1–280.4 Ma), with ages from previous studies spanning from 368 Ma (plastid data, Testo and Sundue, 2016) to 173 Ma (nuclear, Shen et al., 2018).

In contrast with other eusporangiate fern relationships, there were relatively high levels of gene tree–species tree conflict in the placement of Marattiales, but the best-supported topology had Marattiales sister to the leptosporangiate ferns (Figures 2, 3B). This topology is supported in most other large-scale fern phylogenies (Knie et al., 2015; Rothfels et al., 2015; Testo and Sundue, 2016; Kuo et al., 2018a; Qi et al., 2018), although some have found support for Psilotales and Ophioglossales sister to Marattiales (Shen et al., 2018; see also our third codon position analysis, Supplementary Figure 2) or Psilotales and Ophioglossales sister to the leptosporangiates (Figure 3B). The topology in One Thousand Plant Transcriptomes Initiative (2019) also shows substantial conflict in gene trees relative to the species tree here, with relatively equal proportions of gene trees supporting Marattiales sister to Ophioglossales and Psilotales, and leptosporangiates sister to Ophioglossales and Psilotales. We found a Marattialean stem age of 325 Ma (range 316.9–331.5 Ma), which is largely consistent with other findings (plastid: 365 Ma, Testo and Sundue, 2016; nuclear: 329–355 Ma, Rothfels et al., 2015; Qi et al., 2018). Although our sampling in this part of the phylogeny is sparse, the relationships we recovered within Marattiales are consistent with those found by Lehtonen et al. (2020) and May et al. (2020), with *Danaea* and *Ptisana* successively sister to a clade containing *Angiopteris* and *Christensenia*.

#### Gleicheniales and Hymenophyllales

The relationships between Gleicheniales and Hymenophyllales have not been previously resolved and the recovered topologies differed among studies with Hymenophyllales and Gleicheniales either forming a clade (Pryer et al., 2004), or not (Schuettpelz and Pryer, 2007; Testo and Sundue, 2016). Rothfels et al. (2015) found different topologies depending on the phylogenetic reconstruction approach used; using 25 low-copy nuclear loci, they found low support for a grade of Hymenophyllaceae, Gleichenaceae, and Dipteridaceae in their maximum likelihood tree, but recovered a clade of these families using a Bayesian approach. With entire plastome sequences, Kuo et al. (2018a) recovered two topologies with high support, one suggesting a grade of Hymenophyllales and Gleicheniales to the remaining leptosporangiate ferns, and the other suggesting a clade of Hymenophyllales and Gleicheniales.

We found that Hymenophyllaceae, Dipteridaceae, and Gleichenaceae formed a single clade, with Hymenophyllaceae sister to Gleicheniaceae, and Dipteridaceae sister to them (Figures 2, 3C), suggesting that Gleicheniales may not be monophyletic. We identified high levels of gene tree–species tree conflict, with high proportions of gene trees supporting alternative topologies to the inferred species tree (Figure 3C). Short branches between critical nodes may represent a rapid divergence among these lineages (Figure 2 and Supplementary Figure 2), suggesting a role for incomplete lineage sorting. In two trees, we recovered a clade of Gleicheniaceae and Hymenophyllales, with Dipteridaceae sister to the remaining ferns (Supplementary Figure 2, SCO85 AA MSC, MCO AA MSC). Within Hymenophyllaceae, we found support for the two subfamilies recognized by The Pteridophyte Phylogeny Group (2016): Trichomanoideae (*Callistopteris, Cephalomanes*, and *Vandenboschia*) and Hymenophylloideae (*Hymenophyllum*). The intra-familial relationships of these genera are similar to the topology recovered by Ebihara et al. (2006), except that we find *Callistopteris* and *Cephalomanes* are sister rather than a grade.

We found a stem age of Hymenophyllaceae of 271 Ma (range 270.6–273.1 Ma), giving this clade an origin in the Permian, which is consistent with other studies (Schuettpelz and Pryer, 2009; Qi et al., 2018; but see Testo and Sundue, 2016 for a possible Carboniferous origin 345 Ma). Our age for Gleicheniaceae is comparable to previous studies (287–263 Ma; Schuettpelz and Pryer, 2009; Qi et al., 2018, respectively) at around 271 Ma (range 270.6–273.1 Ma), while our age for Dipteridaceae is older than those previously recovered (196.1–239.7 Ma; Schuettpelz and Pryer, 2009; Rothfels et al., 2015; Testo and Sundue, 2016; Shen et al., 2018) at 274 Ma (range 272.9–279.0 Ma), and may reflect differences in topology in the dating analyses, although fossil Dipteridaceae suggest an origin in the early Triassic or late Paleozoic (Choo and Escapa, 2018).

Thus far, samples of Matoniaceae, the third family of Gleicheniales, have been lacking in large-scale nuclear fern phylogenies (Wickett et al., 2014; Rothfels et al., 2015; Qi et al., 2018; Shen et al., 2018; One Thousand Plant Transcriptomes Initiative, 2019) and thus are not included here. Matonianceae is a relatively small family with just four species in two genera (The Pteridophyte Phylogeny Group, 2016), but the omission of this family may alter the recovered topologies. Using plastid data, Pryer et al. (2004) and Schuettpelz and Pryer (2007) recovered Matoniaceae sister to Dipteridaceae; it is possible that additional sampling of this family in future work could help further resolve these relationships with nuclear data.

#### Cyatheales

Due in part to their low rates of molecular evolution (Korall et al., 2010), relationships among taxa in Cyatheales have been difficult to resolve (Korall et al., 2006). We recovered six families recognized by The Pteridophyte Phylogeny Group (2016) falling into two major clades, with one including Culcitaceae, Plagiogyriaceae, and Thyrsopteridaceae, and the other comprised of Cibotiaceae, Cyatheaeceae, and Dicksoniaceae (Figure 2). Our phylogeny agrees with the classification and phylogeny in Rothfels et al. (2015); Qi et al. (2018), and Shen et al. (2018), all of which used nuclear data. In contrast, our topology differs from the plastid-based phylogenies reconstructed by Schuettpelz and Pryer (2007) and Testo and Sundue (2016). Both plastid studies recovered a clade composed of Cyatheaceae and Cibotiaceae, with Dicksoniaceae sister to them, while the nuclear data recovered Dicksoniaceae and Cibotiaceae sister to one another, with Cyatheaceae sister to them. Our analysis of the first and second codon positions recovered Thrysopteridaceae sister to the clade composed of Cyatheaceae, Dicksonianceae, and Cibotiaceae, while our other analyses (including of the third codon position) found Thyrsopteridaceae sister to a clade consisting of Culcitaceae and Plagiogyriaceae (Supplementary Figure 2).

Stem ages of each tree fern family vary among studies (Supplementary Figure 4), likely a product of their abrupt shift to lower rates of molecular evolution compared to other ferns. We estimated that the split between the two major clades of tree ferns occurred around 164 Ma (range 110.8–192.5 Ma), although older (206–176 Ma, Schuettpelz and Pryer, 2009; Testo and Sundue, 2016, respectively) and younger (72–162 Ma, Rothfels et al., 2015; Qi et al., 2018, respectively) ages have been suggested. With a comprehensive sampling of 150 taxa in Cyatheales, Barrera-Redondo et al. (2018) estimated the stem age of Cyatheaceae, by far the largest family in the order, to be around 160 Ma, which is similar to both nuclear (157 Ma, Qi et al., 2018) and plastid (174–168 Ma, Schuettpelz and Pryer, 2009; Testo and Sundue, 2016, respectively) studies, but differs considerably from our estimate of 108.6 Ma (range 102.4–170.5 Ma). In our supplemental analysis including the *S. lepifera* transcriptome and additional fossil calibrations (see Section “Materials and Methods” and Supplementary Figure 6), we recovered a stem age of Cyatheaeceae at 121.1 Ma (range 119.8–124.6 Ma), which is still younger than previous estimates.

#### Polypodiales

Polypodiales is the largest of the fern orders and includes 80% of extant fern diversity (The Pteridophyte Phylogeny Group, 2016). Consistent with other studies (e.g., Testo and Sundue, 2016; Du et al., 2021), we find suborders Lindsaeineae and Saccolomatineae form a clade sister to the rest of Polypodiales. We found that Saccolomatineae was sister to a monophyletic Lindsaeineae (Supplementary Figure 6). Within Lindsaeineae, we recovered Cystodiaceae sister to a clade composed of Lindsaeaceae and Lonchitidaceae (Figure 2 and Supplementary Figure 6). The monophyly and relationship of this clade to the rest of Polypodiales has been recovered by several recent studies (e.g., Schuettpelz and Pryer, 2007; Testo and Sundue, 2016; Qi et al., 2018; Du et al., 2021).

We consistently found Pteridaceae and Dennstaedtiaceae successively sister to the eupolypods, with Pteridaceae sister to Dennstaedtiaceae + the eupolypods (Figure 2 and Supplementary Figure 2). This result was also seen in studies using nuclear loci (Rothfels et al., 2015; Shen et al., 2018; One Thousand Plant Transcriptomes Initiative, 2019), whereas others (Pryer et al., 2004; Schuettpelz and Pryer, 2007; Testo and Sundue, 2016) recovered Dennstaedtiaceae and Pteridaceae successively sister to the eupolypods using plastid loci. Du et al. (2021), however, recovered Dennstaedtiaceae and Pteridaceae as a clade sister to the eupolypods. In Pteridaceae, the recovered major clades correspond to subfamilies as circumscribed in PPG I: Cheilanthoideae, Cryptogrammoideae, Parkerioideae, Pteridoideae, and Vittaroideae. Our estimated ages of Pteridaceae and Dennstaedtiaceae are similar to other studies (185–163 Ma, Schuettpelz and Pryer, 2009; Testo and Sundue, 2016, respectively) at 164 Ma (range 145.7–198.0 Ma) and 155 Ma (range 110.6–175.9 Ma; 325–146 Ma, Rothfels et al., 2015; Testo and Sundue, 2016, respectively), respectively. In agreement with Schuettpelz and Pryer (2009), we place the origin of the eupolypods in the early Cretaceous, at 113 Ma (range 79.2–123.6 Ma), although Du et al. (2021) found an older origin in the Jurassic at 160 Ma.

#### Eupolypods I (Polypodiineae)

In contrast to the relationships recovered in Kuo et al. (2011); Zhang and Zhang (2015), and Testo and Sundue (2016) we recovered Hypodematiaceae as sister to the rest of the eupolypods I (Figure 2), in agreement with Qi et al. (2018); Shen et al. (2018), and Du et al. (2021). Interestingly, Schuettpelz and Pryer (2007) and Rothfels et al. (2015) found that Hypodematiaceae and Didymochlaenaceae formed a clade, which was sister to the rest of the eupolypods I. Our analyses recovered Lomariopsidaceae and Nephrolepidaceae as successively sister to the remaining families in the eupolypods I (Figure 2), which is consistent with Testo and Sundue (2016); Qi et al. (2018), Shen et al. (2018), and Du et al. (2021), although Schuettpelz and Pryer (2007) found Lomariopsidaceae and Nephrolepidaceae form a clade rather than a grade.

The monophyly of Tectariaceae had varying levels of support throughout our analyses (Supplementary Figure 2). Some analyses (SCO85 AA ML, SCO60 NT ML), identified *Pteridrys cnemidaria* as sister to a clade consisting of Tectariaceae, Oleandraceae, Davalliaceae, and Polypodiaceae. Others (SCO75 AA MSC, SCO60 AA MSC, MCO NT MSC, MCO AA MSC, codon positions one and two) recovered a clade consisting of *Pteridrys* and *Tectaria* sister to a clade of *Arthropteris*, Oleandraceae, Davalliaceae, and Polypodiaceae. Despite the different topologies recovered in our analyses, several recent studies have recovered Tectariaceae to be monophyletic (including all three genera sampled here) (Liu et al., 2014; Zhang et al., 2016; Zhou et al., 2018) although Zhou et al. (2018) suggest a new family (Pteridryaceae) be recognized. While Du et al. (2021) separate Tectariaceae from Pteridryaceae (Zhou et al., 2018) and Arthopteridaceae (suggested by Liu et al., 2013, but not recognized by The Pteridophyte Phylogeny Group, 2016), they found the three families form a clade sister to the remaining eupolypods I. Results for the remaining families within the eupolypods I (e.g., Dryopteridaceae and Polypodiaceae) were relatively consistent with those from previous studies and The Pteridophyte Phylogeny Group (2016).

#### Eupolypods II (Aspleniineae)

The relationships within the eupolypods II have been difficult to resolve due to heterogeneity in rates of molecular evolution among families and the rapid radiation of lineages in the group (e.g., Rothfels et al., 2012). In particular, the placement of Aspleniaceae varied amongst our analyses, with concatenated ML analyses finding Cystopteridaceae sister to the rest of the eupolypods II, with Aspleniaceae and Diplaziopsidaceae forming a clade nested within the eupolypods II (see Supplementary Figure 2), or a clade of Aspleniaceae and Diplaziopsidaceae sister to the rest of the eupolypods II (SCO85 NT MSC), although most MSC analyses resolved Aspleniaceae as sister to the rest of the eupolypods II with the remaining families forming a grade (Supplementary Figure 2). The latter relationship (depicted in Figure 2) reflects the result found in Testo and Sundue (2016), although Rothfels et al. (2012) resolved relationships of the eupolypods II by assessing and addressing model misspecifications in a Bayseian framework, resulting in Aspleniaceae nested within the eupolypods II rather than sister to the rest. They did, however, only use plastid data for these analyses; further work by Rothfels et al. (2015) using nuclear data support the topology of the eupolypods II in Rothfels et al. (2012). Using full plastome sequences, Wei et al. (2017) and Du et al. (2021) found Cystopteridaceae sister to the rest of the eupolypods II, with Aspleniaceae, Desmophlebiaceae (not sampled here), Hemidictyaceae (not sampled here), Diplaziopsidaceae, and Rhachidosoraceae forming a clade nested within eupolypods II (RHADD clade *sensu* Du et al., 2021). However, other nuclear datasets and particularly differing analyses, have contradicted this result (Qi et al., 2018; Shen et al., 2018), with concatenation-based ML analyses supporting the RHADD clade, while MSC analyses placed Aspleniaceae sister to the rest of the eupolypods II.

### Challenges in Fern Phylogenetics

Despite recent advances in fern systematics and our ability to use thousands of markers throughout the genome to reconstruct phylogenetic relationships, challenges certainly remain in fully resolving the fern tree of life. In particular, we address the difficulties posed by lineage-specific rate heterogeneity, nuclear-plastid incongruence, and polyploidy.

#### Lineage-Specific Rate Heterogeneity

Differences in the rates of molecular evolution in different clades have made resolving relationships and estimating divergence times difficult across the entire tree of life (e.g., Beaulieu et al., 2015; Carruthers et al., 2020). In ferns, some clades of special interest regarding rate heterogeneity are Aspleniaceae (Rothfels et al., 2012; Wei et al., 2017), Cyatheales (Korall et al., 2010), Hymenophyllales (Schuettpelz and Pryer, 2006), Marattiales (Soltis et al., 2002), Osmundales (Rothfels et al., 2015), and vittaroid ferns (Rothfels and Schuettpelz, 2014; Grusz et al., 2016; see Supplementary Figure 2). Compared to the rest of the ferns, Cyatheales, Marattiales, and Osmundales have decelerated rates of molecular evolution (e.g., Soltis et al., 2002; Korall et al., 2010; Rothfels et al., 2015). Increased longevity and generation time has been posited as explanations for the rate heterogeneity seen in other plants (e.g., Gaut et al., 1992); for example, in angiosperms, annuals have been shown to have higher rates of molecular evolution than perennials and arborescent plants (Smith and Donoghue, 2008). A similar pattern has emerged in ferns; deceleration of molecular evolution has been linked to the evolution of arborescence in tree ferns (Korall et al., 2010). The cause for decreased rates in Osmundales and Marattiales may be linked to their long generation times, with individual clones of *Osmunda* suggested to live more than 1000 years (Wagner et al., 1978). In contrast, Aspleniaceae, Trichomanoideae, and vittaroid ferns have experienced accelerated rates of molecular evolution (Schuettpelz and Pryer, 2006; Rothfels et al., 2012; Rothfels and Schuettpelz, 2014; Grusz et al., 2016; Testo and Sundue, 2016). Shifts in life history, morphology, and/or ecological niche may explain changes in their rates of molecular evolution. For example, the biology of vittaroid ferns differs from their closest relatives, as they are tropical, simple-leaved mostly epiphytic plants (as opposed to arid-adapted, dissected-leaved, epipetric plants) and experience a 4.3 times faster rate of molecular evolution than cheilanthoid ferns (Rothfels and Schuettpelz, 2014). Given the range in rates across the fern phylogeny and the implications for divergence time estimation and phylogeny reconstruction, lineage-specific rate heterogeneity is one of the biggest challenges in modern fern phylogenetics.

#### Nuclear–Plastid Incongruence

The vast majority of plant (and specifically fern) systematics to date has relied on plastid loci (e.g., Chase et al., 1993; Pryer et al., 2001, 2004; Schuettpelz and Pryer, 2007; Testo and Sundue, 2016). However, the plastid is a maternally inherited, single linkage group, and therefore only captures part of evolutionary history (Lynch, 2007; but see Gonçalves et al., 2019 for a suggestion that all the loci in the plastid are not truly linked). One of the biggest challenges for nuclear phylogenetics in ferns has been working with single-copy (or low-copy) loci. Due to multiple rounds of polyploidy and tandem duplications, it is unlikely that any locus is truly single-copy in all fern genomes (although most gene families quickly revert back to single copy, De Smet et al., 2013; Li et al., 2016). This has made it particularly challenging to identify and sequence single- or low-copy nuclear genes throughout ferns (Rothfels et al., 2013). Furthermore, resolving recalcitrant relationships among ferns requires several unlinked, bi-parentally inherited markers, which cannot be accomplished with just plastid markers. Recent advances in computational tools have made it possible to analyze multi-copy loci datasets such as ASTRAL-PRO (Zhang et al., 2020). Large-scale work with the nuclear genome has been relatively recent, but the results have been surprisingly congruent with plastid phylogenies (Wickett et al., 2014; Rothfels et al., 2015; Qi et al., 2018; Shen et al., 2018; One Thousand Plant Transcriptomes Initiative, 2019). The same problematic nodes and relationships in plastid phylogenies have been replicated in nuclear phylogenies (Figure 3), though, as discussed at length above, some differences are consistent between plastid and nuclear phylogenies (e.g., the sister group to the eupolypods, Figure 3D).

#### Polyploidy

Manton’s (1950) work revealed that ferns have high chromosome numbers (and later large genome sizes, e.g., Clark et al., 2016) and subsequent studies have confirmed that polyploidy is a prevalent phenomenon in extant ferns. The extent to which polyploidy has occurred throughout the phylogeny, particularly at deeper nodes, has only recently been explored (Huang et al., 2019; One Thousand Plant Transcriptomes Initiative, 2019; Li and Barker, 2020). Paralogs from WGDs make it difficult to assess orthology across multiple species using only a reciprocal homology search algorithm, especially when duplications are recurrent. Several clustering programs (e.g., Li et al., 2003; Yang and Smith, 2014; Emms and Kelly, 2015, 2019) and downstream phylogenetic methods (e.g., Zhang et al., 2020) have been developed to account for the presence of paralogs in phylogenomic datasets. While polyploidy is a prevalent process in ferns that can make it difficult to reconstruct evolutionary histories, new methods and the advent of high-throughput sequencing techniques have been instrumental for tackling this challenge.

### Phylogenetic Placement of Whole Genome Duplications

Polyploidy has long been recognized as an important evolutionary mechanism in plants (Manton, 1950; Stebbins, 1950; Klekowski and Baker, 1966), although our view of the role of polyploidy in plant evolution has shifted dramatically throughout the past century (reviewed by Soltis et al., 2014b). One of the major debates in the polyploid literature is the role of genome duplications in diversification. Polyploid plants have been suggested to diversify at faster rates than diploids (Soltis et al., 2014a; Tank et al., 2015; Landis et al., 2018; Román-Palacios et al., 2020; but see Mayrose et al., 2011; Arrigo and Barker, 2012; Mayrose et al., 2015 for contrasting results). Although we do not address diversification here, it is of note that the two inferred WGDs shared by the largest number of taxa are at the base of the most diverse fern clades. By placing WGDs on the phylogeny constructed by Qi et al. (2018); Huang et al. (2019) found that clades of ferns that underwent two or more WGD events had higher diversification rates than other clades. They propose that the three rounds of fern radiations (Rothwell, 1987; Rothwell and Stockey, 2008; Schuettpelz and Pryer, 2009) correspond to WGDs at the base of the leptosporangiates, core leptosporangiates, and Polypodiales (Huang et al., 2019). While this is an enticing possibility, further testing will be required to explore these patterns, such as running these analyses with the varying hypotheses of the placement of WGDs in ferns posited by Huang et al. (2019), One Thousand Plant Transcriptomes Initiative (2019), and this study. Furthermore, generating a phylogeny with more comprehensive sampling or placing WGDs on an existing tree with extensive sampling may be more appropriate for these diversification analyses. Additional work will be required to determine if there is a relationship between polyploidy and diversification in ferns.

Paralog age distributions (K_*S*_ plots) have been the primary tool of choice for inferring WGDs; briefly, the synonymous distances between genes within a gene family (often defined by a clustering algorithm such as OrthoMCL, Li et al., 2003) are calculated and a histogram of the frequency or count of these distances is plotted. Peaks in the K_*S*_ distribution are interpreted as the result of large-scale duplication events (i.e., polyploidy; Lynch and Conery, 2000; Vanneste et al., 2013). By rigorously testing the applicability of K_*S*_ plots to infer ancient WGDs (paleopolyploidy events), Tiley et al. (2018) found that K_*S*_ plots should be used primarily as a hypothesis-building tool and should be supplemented with other lines of evidence. One of the biggest challenges in using paralog K_*S*_ values relative to speciation events (ortholog divergence) to infer WGDs is lineage-specific rate heterogeneity. Several methods have been developed to account for these rate differences (e.g., Barker et al., 2008; Sensalari et al., 2022) to yield corrected K_*S*_ values, which may reveal different phylogenetic placements of WGDs. Interestingly, when we compared WGD inferences from corrected and uncorrected K_*S*_ most of the WGD inferences had identical phylogenetic placements (12 identical phylogenetic placements out of 20, including LONCH; Figure 4). As expected, correcting K_*S*_ in lineages with shifts in molecular evolutionary rates such as the tree ferns and Osmundaceae resulted in different placements of the WGD; however, phylogenomic approaches generally supported the uncorrected K_*S*_ placements, rather than the corrected K_*S*_ placement. Even after correcting for rate heterogeneity, there were some cases where K_*S*_ between one-to-one orthologs were ambiguous (Figure 4 and Supplementary Figures 8, 9), likely due to rapid divergences (e.g., GLEI, DIPT.1) or differences in rates of molecular evolution (e.g., CYATH.1-3).

Recent advances in phylogenomic methods have been applied to placing WGDs on a species tree (e.g., Li et al., 2015; McKain et al., 2016; Li Z. et al., 2018). These methods examine the proportion of gene trees with a shared duplication event at the nodes of a species tree; many shared duplications support a large-scale duplication event. One of the limitations to MAPS is that the software requires a ladderized tree as input, which requires users to subsample their phylogeny. To test whether there was an effect of the sample choice, we ran MAPS using each *P. nudum* sample in the PSIL analysis (see Supplementary Appendix 5), which resulted in nearly identical proportions of gene duplicated gene trees at each node in the species tree (e.g., <1% difference). Similarly, using different lineages may impact the inference of WGDs. We tested whether this may impact our results by using different *Equisetum* species in the EQUI analysis (see Supplementary Appendix 5) and again found minimal changes to the proportion of duplicated gene trees at each node and no change in the inference of the WGD event. While we did not find that taxon selection had an impact on the inference of the phylogenetic placement of WGDs, further work should be considered to determine if and how this process can affect inferences. Both K_*S*_ plots and gene tree-based methods are accessible for data generated from transcriptomes. Full genome assemblies may further be interrogated to assess duplication events using synteny (i.e., gene order; Li F. W. et al., 2018; Krabbenhoft et al., 2021). To infer WGDs in this study, we used a combination of synonymous distance between paralogs within species and one-to-one orthologs between species, a phylogenomic method implemented in MAPS and rigorous statistical testing to place WGDs on the fern phylogeny. In general, these lines of evidence were consistent.

Overall, the placement of WGDs identified here was similar to previous estimates (Huang et al., 2019; One Thousand Plant Transcriptomes Initiative, 2019). Of the 18 large-scale WGDs we inferred, 12 had identical placements in the phylogeny produced by Huang et al. (2019). Below we discuss events which are novel or whose placement differed between our study and previous inferences, starting at the base of phylogeny. We emphasize that the inferred events should be treated as hypotheses and require further study, including whole genome analyses.

In contrast with One Thousand Plant Transcriptomes Initiative (2019), we did not find a duplication shared by all ferns, although the placement of our duplications suggest that nearly all ferns sampled have a polyploid history (Figure 2). The placement of the duplication at the base of Ophioglossales found in One Thousand Plant Transcriptomes Initiative (2019) was not recovered in our study, although a WGD shared by *Ophioglossum* was identified (OPHIO.2). Unlike Huang et al. (2019), we did not find that this duplication was shared by *Ophioglossum* and *Ophioderma*; rather we found that *Ophioderma* underwent a separate duplication event (OPHIO.3). These inferences are supported by several lines of evidence including corrected and uncorrected K_*S*_ values and MAPS. In Psilotales, we identified a novel duplication in *Tmesipteris* relative to *Psilotum*, with support from both K_*S*_ analyses, MAPS, and karyotypes. All three studies agree on the placement of a WGD at the base of the leptosporangiates excluding Osmundales (LEPTO). While Huang et al. (2019) and One Thousand Plant Transcriptomes Initiative (2019) found that Osmundales underwent a separate duplication event our analyses suggested that the uncorrected K_*S*_ inference was ambiguous, the corrected K_*S*_ suggested an event shared by all leptosporangiate ferns, and MAPS failed to identify a significant difference in the observed and null distribution of duplicated gene trees. While One Thousand Plant Transcriptomes Initiative (2019) did not identify duplication events in Hymenophyllales and Gleicheniales (only two taxa were included from these groups in their phylogeny), our findings are similar to Huang et al. (2019), although we found novel WGDs on the branches leading to *Dipteris* (DIPT2) and Trichomanoideae (HYMN), both of which had taxa that were sampled in Huang et al. (2019).

Unlike One Thousand Plant Transcriptomes Initiative (2019), we did not infer a WGD at the base of Schizaeales, and the inferred event may instead be representative of LEPTO. The uncorrected K_*S*_ plots for *Azolla* spp. were ambiguous but the corrected K_*S*_ clearly identified the inferred WGD shared by *Azolla* based on MAPS; syntenic analysis further support this placement (Li F. W. et al., 2018). Although both Huang et al. (2019) and One Thousand Plant Transcriptomes Initiative (2019) identified events on the backbone of the phylogeny either shared by Cyatheales + Polypodiales or Salviniales, Cytheales, and Polypodiales, respectively, we did not identify a WGD event occurring at either of these locations. Within Cyatheales, Huang et al. (2019), One Thousand Plant Transcriptomes Initiative (2019), and Huang et al. (2022) identified a WGD shared by *Alsophila*, although our analyses suggests instead it is shared by Cibotiaceae, Cyatheaceae, and Dicksoniaceae (CYATH.3). Although Huang et al. (2022) generated a chromosome-scale genome for *A. spinulosa*, they used the same approaches used here (K_*S*_ analyses and MAPS), although further syntenic evidence will be required to verify the placement of CYATH.3, especially given the uniformly high chromosome numbers in Cyatheales (see Section “Results”) and slower rates of molecular evolution in the tree ferns. Along the backbone of the phylogeny, we inferred a WGD shared by Polypodiales excluding Lindsaeineae and Saccolmatineae (POLY), the placement of which agrees with One Thousand Plant Transcriptomes Initiative (2019). Within Pteridaceae, we found one shared event at the base of the vittaroid ferns (PTER.3) that was not identified in One Thousand Plant Transcriptomes Initiative (2019). We did not find evidence of the WGD on the branch leading the eupolypods, which was recovered in Huang et al. (2019). Although we did identify a significantly greater proportion of duplicated gene trees than in the null distribution at the MRCA of the eupolypods, the observed distribution was not consistent with positive simulations of a WGD.

### Gene Retention Following Whole Genome Duplications and Large-Scale Duplications

As with most other plants, we found that the rate of duplicate gene retention is low following paleopolyploidy or large-scale gene duplications, with an average of around 11.97% of genes remaining in duplicate. On average, duplicate genes have a retention rate around 10% in plants (Tiley et al., 2016), but vary depending on the age of the duplication (Li et al., 2016), with up to 76.3% of genes duplicated in *Glycine max* (Tiley et al., 2016) which underwent a recent duplication event. Genomes which have undergone more recent duplications tend to show higher retention rates than older duplications, with the proportion of the gene families duplicated rapidly decaying with the age of events (Li et al., 2016). Given that the gene families we analyzed were not restricted to “core gene families” as in Li et al. (2016) and that the signal of WGDs becomes diluted as synonymous divergence increases, we were not able to replicate these findings in ferns. While our method of identifying paralogs from WGDs using K_*S*_ plots has been described and used before (e.g., Li Z. et al., 2018), it is important to note that other types of duplication events other than WGDs may contribute paralogs to the distribution. In some cases, particularly in older WGDs, peaks may be difficult to distinguish from the background processes affecting duplicated genes. Although we assigned paralogs to WGD events based on their probability of belonging to certain components in mixture models (see Section “Materials and Methods”), we may be including other duplicated genes that may not have arisen from WGDs.

**FIGURE 5:**
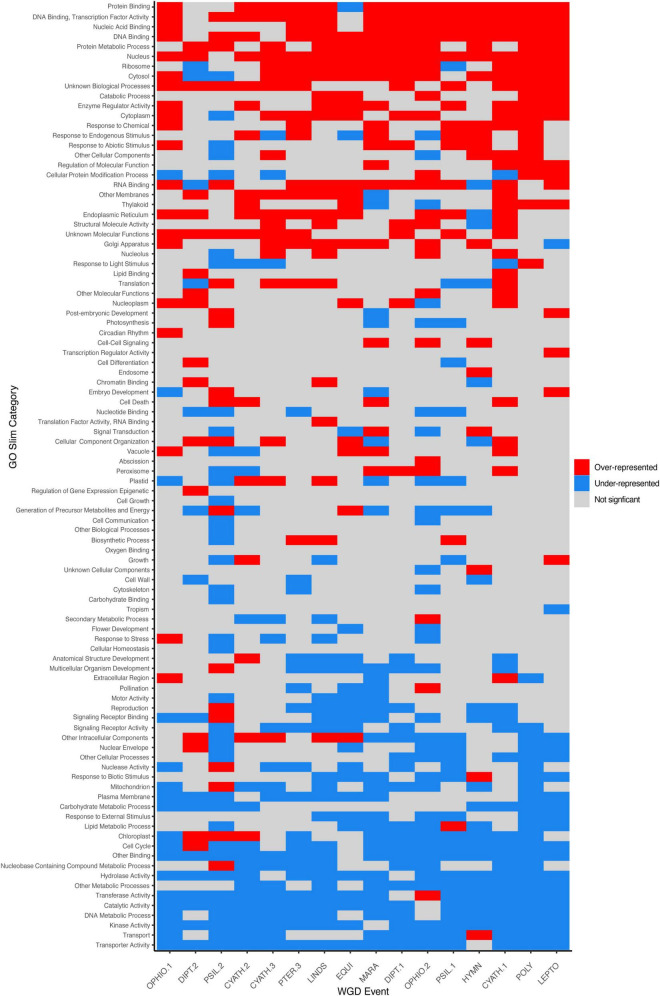
Gene retention in ferns following large-scale duplications is biased. Gene ontology (GO) Slim composition of retained duplicates (paralogs) from 17 of the 18 large-scale whole genome duplications (WGDs) identified in this study are shown, where categories that are over-represented in paralogs are in red (Chi-squared residuals > 2) and under-presented as blue (Chi-squared residuals < -2). Terms with non-significant residuals are gray. Events are sorted by ascending median Ks values. Note that SALV is not shown here; while MAPS identified a significant proportion of duplicated gene trees, uncorrected K_*S*_ plots were ambiguous, although a WGD was identified with syntenic analyses in Li F. W. et al. (2018).

While most duplicated genes are rapidly lost (Lynch and Conery, 2000), the functions and types of genes retained in duplicate following independent WGD events are similar (e.g., Barker et al., 2008; Freeling, 2009; Li et al., 2016; Li Z. et al., 2018; Figure 5). According to the DBH (Papp et al., 2003), the loss of duplicated copies of some but not all partners in an interaction would alter the stoichiometry of the product/network and therefore the ultimate function may be disrupted. Compared to full transcriptomes, we found that genes retained in duplicate have functions that are over-represented in binding processes (DNA binding, transcription factor activity, nucleic acid binding, protein binding, RNA binding, nucleotide binding), responses to stimuli (response to chemical, response to endogenous stimulus, response to abiotic stimulus), and certain organelles involved in gene and protein production and processing (nucleus, ribosome, endoplasmic reticulum, Golgi apparatus) (Figure 5). Binding processes (DNA binding, RNA binding, nucleic acid binding) were also found to be over-represented in paralogs retained in hexapods (Li Z. et al., 2018) and intermediate-age duplicates in angiosperms (Li et al., 2016). Processes involved in transport (transporter activity, transport, nuclear envelope), organelles derived from endosymbiotic events (mitochondrion, chloroplast), and signaling (signaling receptor binding and activity) were under-represented in paralogs (Figure 5). Similar patterns of gene loss are again seen in hexapods (transport, mitochondrion, nuclear envelope, Li Z. et al., 2018), and angiosperms (transport, transporter activity, mitochondrion, Li et al., 2016). While lineage-specific variation in patterns of gene retention is present (see below), some patterns appear to be conserved over deep evolutionary time among the kingdoms of life.

While the overall pattern of gene functions is similar across independent duplication events, there were several instances that suggest that other factors may also drive lineage-specific differences in retained duplicates. For example, retained duplicates following the inferred Psilotales (PSIL.1) and *Tmesipteris* (PSIL.2) events were over-representative of genes involved with endosymbiotic organelles (chloroplast, mitochondrion) and signal receptor binding, while duplicates from most other events were under-represented in those categories (Figure 5). Similarly, genes with functions related to the ribosome and cytosol were under-represented in retained duplicates following the *Dipteris* event (DIPT.2) (Figure 5), although this may be due to low sample size. In Asteraceae, Barker et al. (2008) found several GO Slim categories that were under-represented in duplicates following independent WGDs which we found to be over-represented in fern duplicates (e.g., DNA or RNA binding, nucleus) whereas others were over-represented (e.g., cytosol, protein metabolic process) or under-represented (e.g., chloroplast) in both analyses. Furthermore, the age of the inferred WGD may impact the function of gene sets retained, as in angiosperms (Li et al., 2016); for instance, genes with functions related to translation and metabolic processes were over-represented in older WGDs, but under-represented in more recent events and single-copy gene families. Gene function may therefore be an important factor in the long-term survival of duplicated genes.

While genic diploidization (i.e., the process of removal and loss of genes by molecular mechanisms, Li et al., 2021) is clearly occurring in ferns, perhaps through pseudogenization/gene silencing as hypothesized by Haufler and Soltis (1986), cytological diploidization may be slow to follow. Unlike other plants, chromosome number and genome size are positively correlated in ferns (Clark et al., 2016) and, taken with the relative stasis in genome size across ferns, may suggest that chromosomes are retained following WGDs rather than lost during genomic reorganization in angiosperms. A similar pattern has been observed in the catostomid fish *Myxocyprinus asiaticus*, which shows remarkable genome subgenome stability and retained synteny over 50 MY following a WGD (Krabbenhoft et al., 2021). In contrast, one subgenome tends to dominate in polyploid plants (Alger and Edger, 2020) and rearrangements drastically alter gene order and retention of synteny (Zhao and Schranz, 2018; but see VanBuren et al., 2020 for an example of subgenome stability in plants following a WGD 1 Ma). Whether similar patterns of genome reorganization or stability are present in ferns remains uncertain and an active area of research.

Importantly, transcriptomes are temporal and spatial “snapshots” of gene expression. Many of the transcriptomes used in this study were derived from young leaf material, although some are from other tissue types (e.g., fertile pinnae, gametophytes). Not all genes in the genome will be expressed in every tissue and therefore transcriptomes from single tissues will likely not represent the entirety of gene-space in a genome. The presence or absence of genes in a transcriptome may not necessarily correlate to the presence or absence of that gene in the genome but could rather be a product of differences in expression between tissues and time. The analysis presented here is one of the first to tackle functional gene retention following WGDs in ferns, but additional analyses will be needed to explore whether these patterns of gene retention are specific to transcriptomic study. As new genome assemblies are becoming available (e.g., *Adiantum, Alsophila, Ceratopteris*) patterns of gene retention should be further explored in more complete gene spaces.

## Conclusion

Ferns are a ubiquitous part of global floras and occupy a pivotal evolutionary position sister to seed plants, yet genomic resources for this group are lacking. Using publicly available transcriptome data, we addressed fundamental questions about the evolution of ferns, particularly the nuclear phylogenetic backbone, the placement and number of WGDs along the phylogeny, and the fates of duplicated genes following WGDs. Despite using thousands of loci, areas of the fern phylogeny remain contentious, including the sister group to the leptosporangiate ferns, the relationships among Gleicheniales and Hymenophyllales, the sister group to the eupolypods, and the placement of Aspleniaceae within the eupolypods II. We recovered a number of paleopolyploidy events throughout the phylogeny and found that functions of genes retained in duplicate following polyploidy are largely convergent, with duplicate genes of similar function retained between events. Given the high number of polyploidy events in ferns, questions related to fern evolution must account for WGDs. As sequencing costs continue to decrease and genomics becomes more accessible, ferns will no longer remain one of the final frontiers in plant genomics.

## Data Availability Statement

Publicly available datasets were analyzed in this study. This data can be found here: https://github.com/jessiepelosi/ferntxms.

## Author Contributions

JP planned and designed the research. JP and EK analyzed the data. All authors wrote and revised the manuscript, read, and approved the final manuscript.

## Conflict of Interest

The authors declare that the research was conducted in the absence of any commercial or financial relationships that could be construed as a potential conflict of interest.

## Publisher’s Note

All claims expressed in this article are solely those of the authors and do not necessarily represent those of their affiliated organizations, or those of the publisher, the editors and the reviewers. Any product that may be evaluated in this article, or claim that may be made by its manufacturer, is not guaranteed or endorsed by the publisher.

## Footnotes

1
www.bioinformatics.babraham.ac.uk/projects/fastqc/
