# Supplementary material for: Phylotranscriptomics Illuminates the Placement of Whole Genome Duplications and Gene Retention in Ferns
Source: Front Plant Sci. 2022 Jul 14;13:882441. doi: 10.3389/fpls.2022.882441 (PMC9330400; doi:10.3389/fpls.2022.882441)
Supplement: Supplementary file 1 [file Data_Sheet_1.PDF]

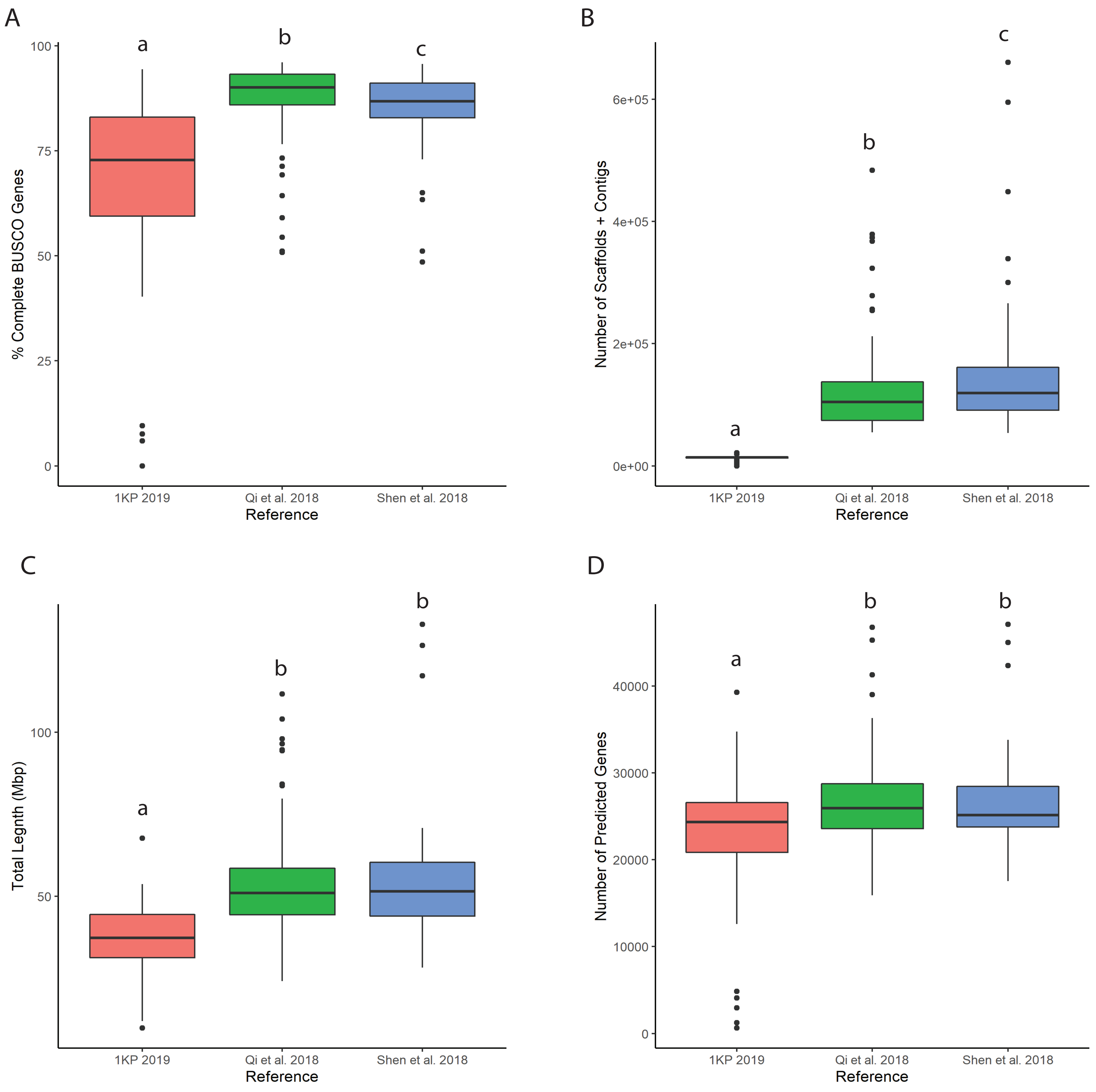

Supplemental Figure 1. Boxplots of metrics comparing transcriptome assemblies from the studies used in this paper; A) BUSCO completeness, B) number of scaffolds and contigs, C) transcriptome length, and D) number of predicted genes. Significantly different groups are denoted with letters.

FigS2A. Maximum likelihood species tree from concatenated SCO85 nucleotide dataset. Branch supports are given as ultra-fast bootstrap support, branch lengths are proportional to number of substitutions per site.

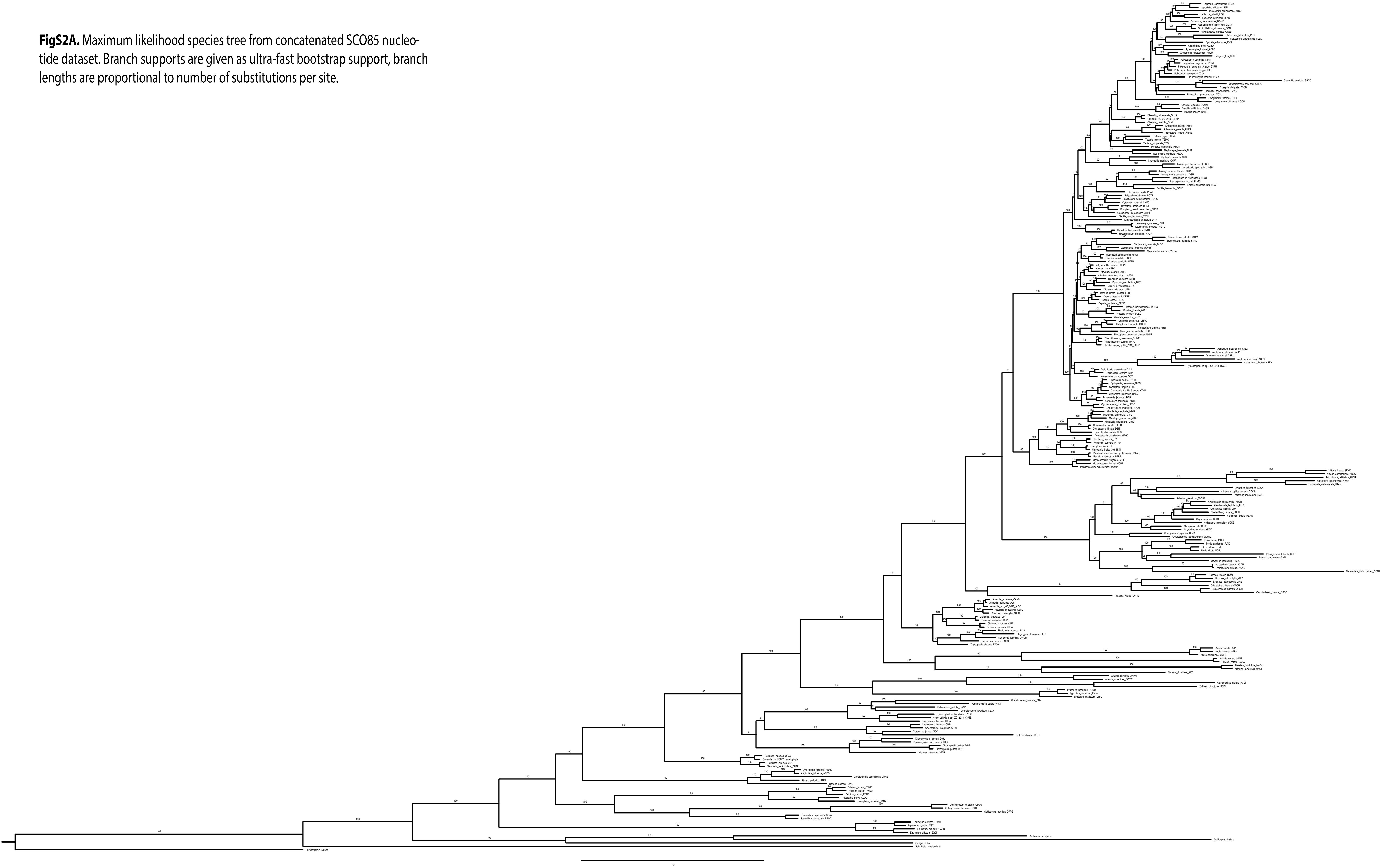

FigS2B. Maximum likelihood species tree from concatenated SCO85 amino acid dataset. Branch supports are given as ultra-fast bootstrap support, branch lengths are proportional to number of substitutions per site.

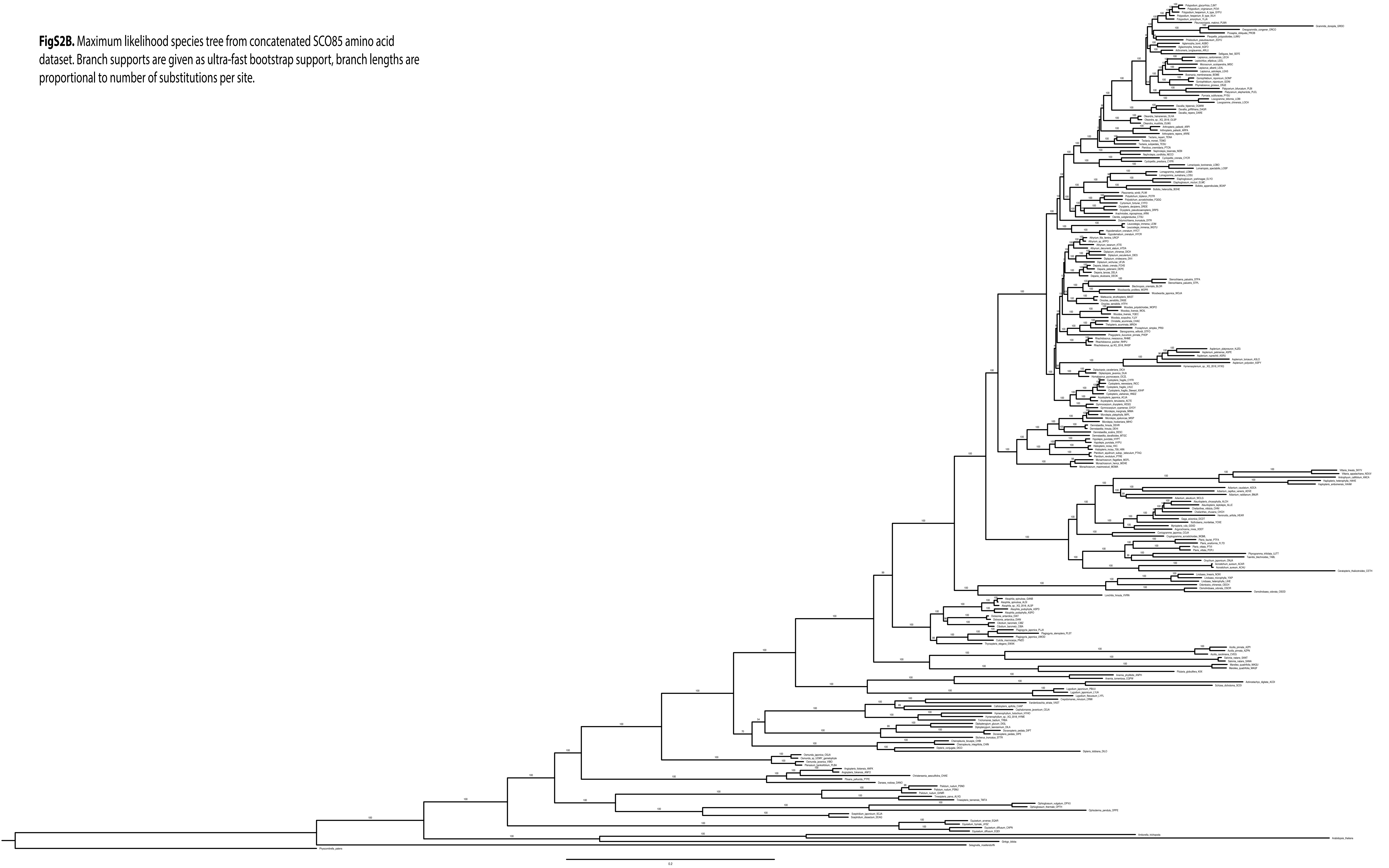

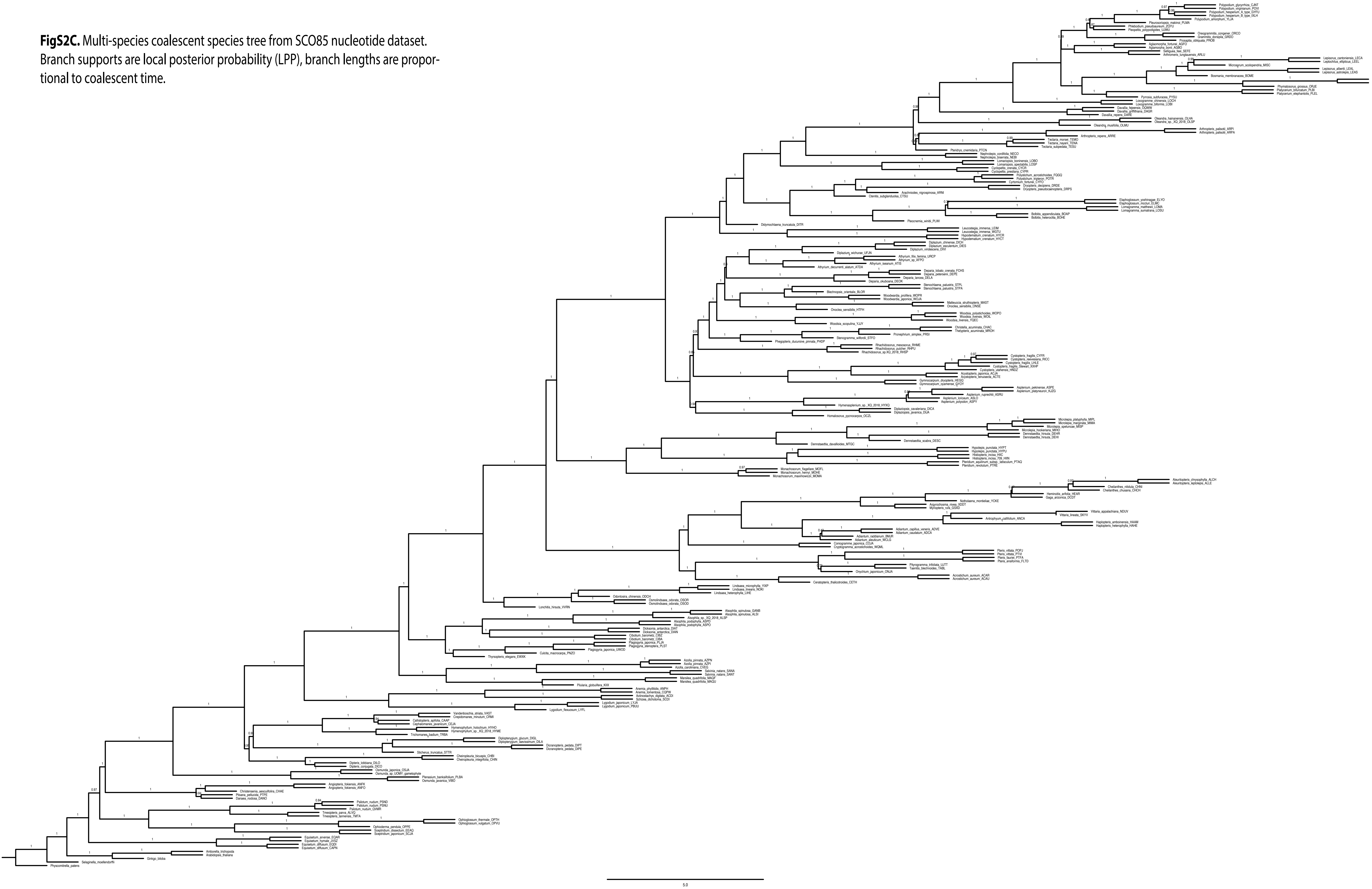

FigS2C. Multi-species coalescent species tree from SCO85 nucleotide dataset. Branch supports are local posterior probability (LPP), branch lengths are proportional to coalescent time.

FigS2D. Multi-species coalescent species tree from SCO85 amino acid dataset. Branch supports are local posterior probability (LPP), branch lengths are proportional to coalescent time.

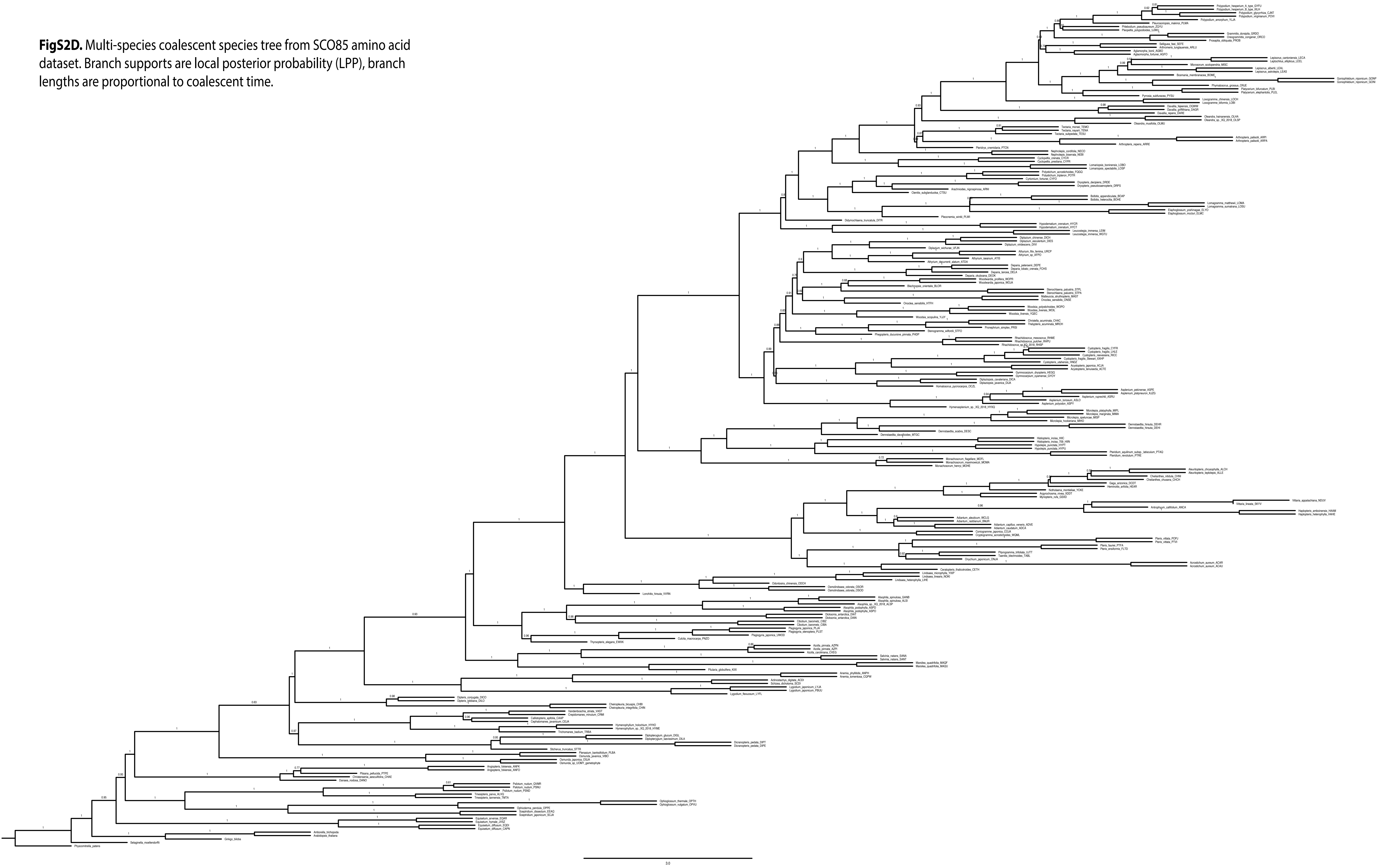

FigS2E. Maximum likelihood species tree from concatenated SCO75 nucleotide dataset. Branch supports are given as ultra-fast bootstrap support, branch lengths are proportional to number of substitutions per site.

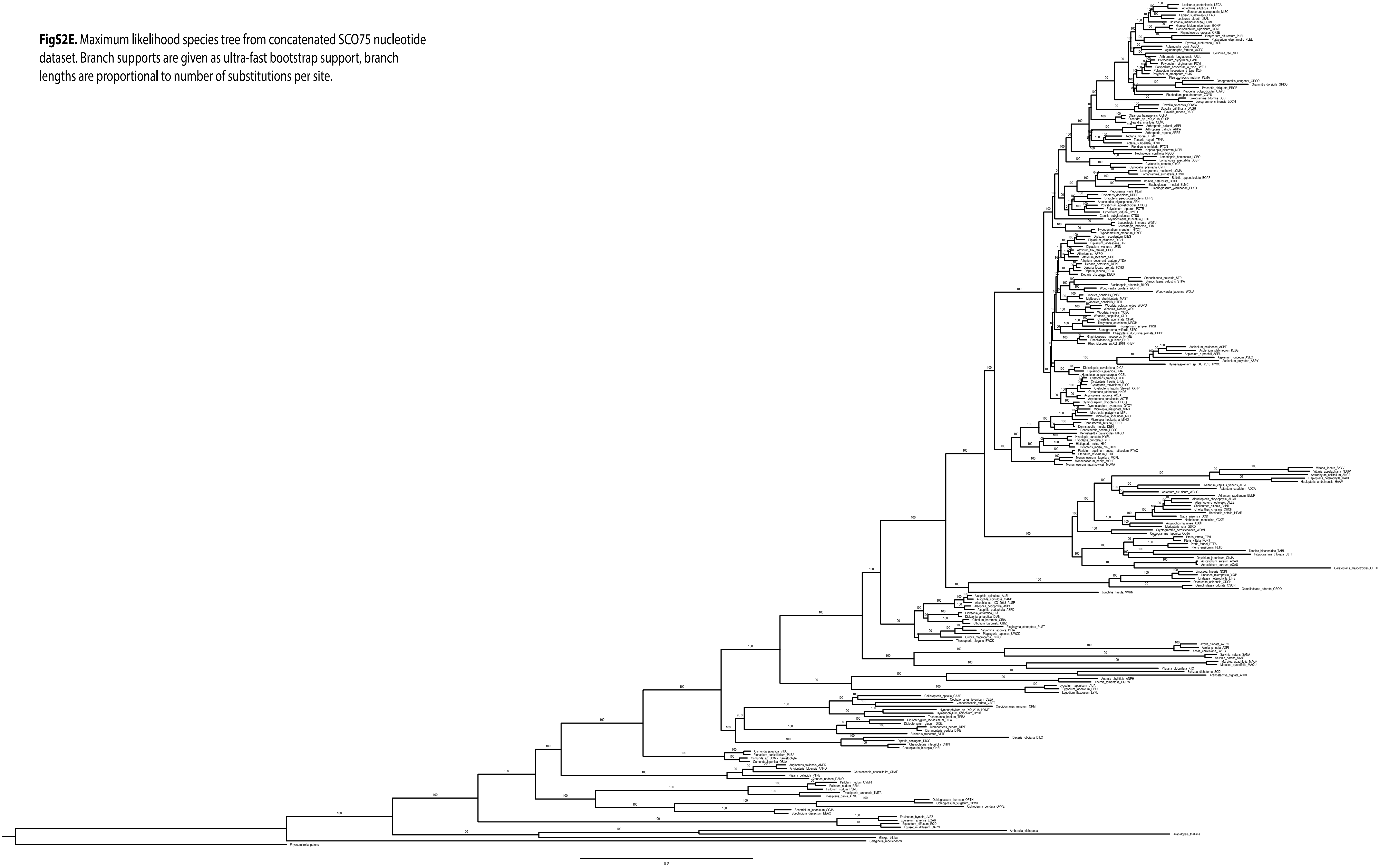

FigS2F. Maximum likelihood species tree form concatenated SCO75 amino acid dataset. Branch supports are given as ultra-fast bootstrap support, branch lengths are proportional to number of substitutions per site.

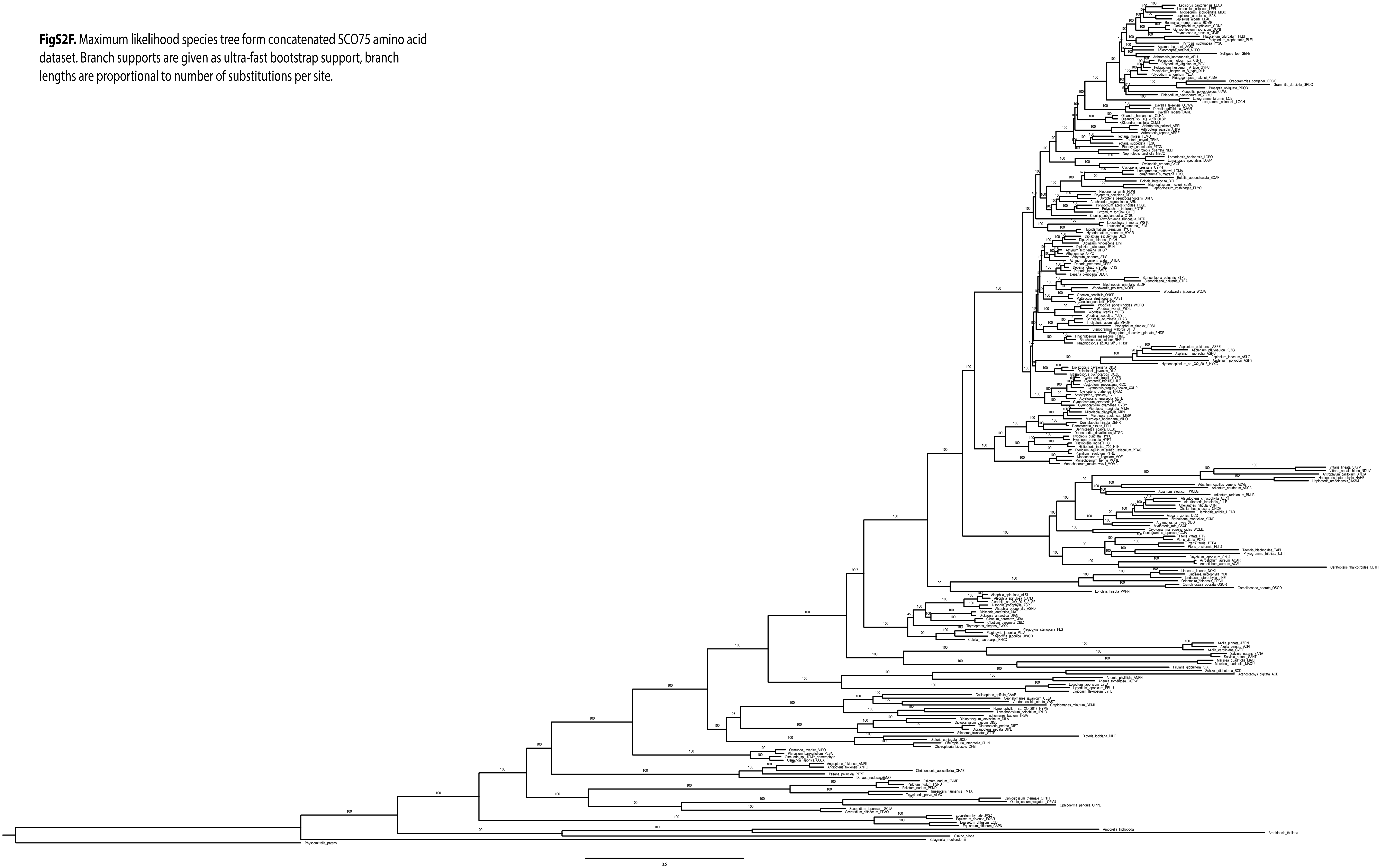

FigS2G. Multi-species coalescent species tree from SCO75 nucleotide dataset. Branch supports are local posterior probability (LPP), branch lengths are proportional to coalescent time.

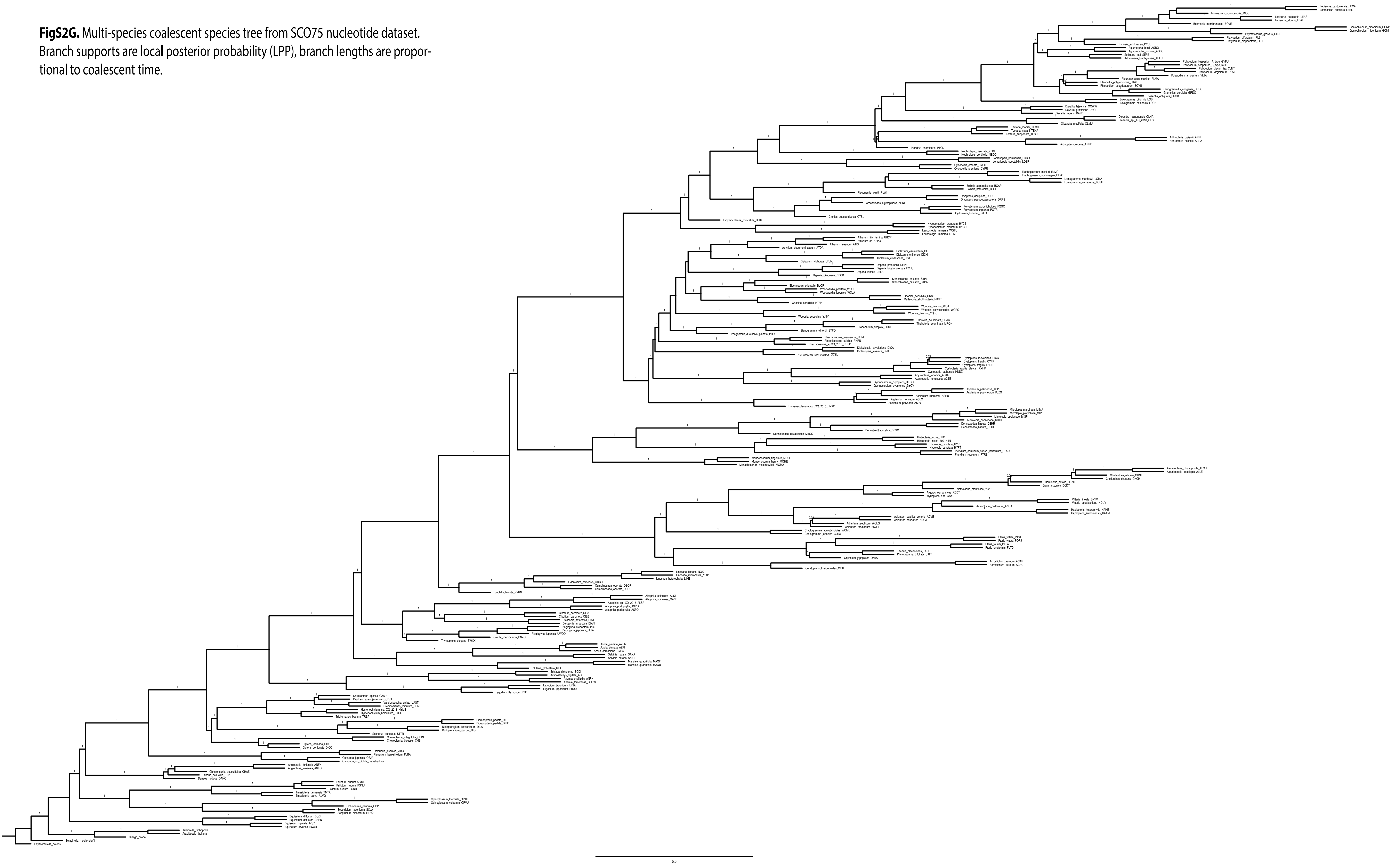

FigS2H. Multi-species coalescent species tree from SCO75 amino acid dataset. Branch supports are local posterior probability (LPP), branch lengths are proportional to coalescent time.

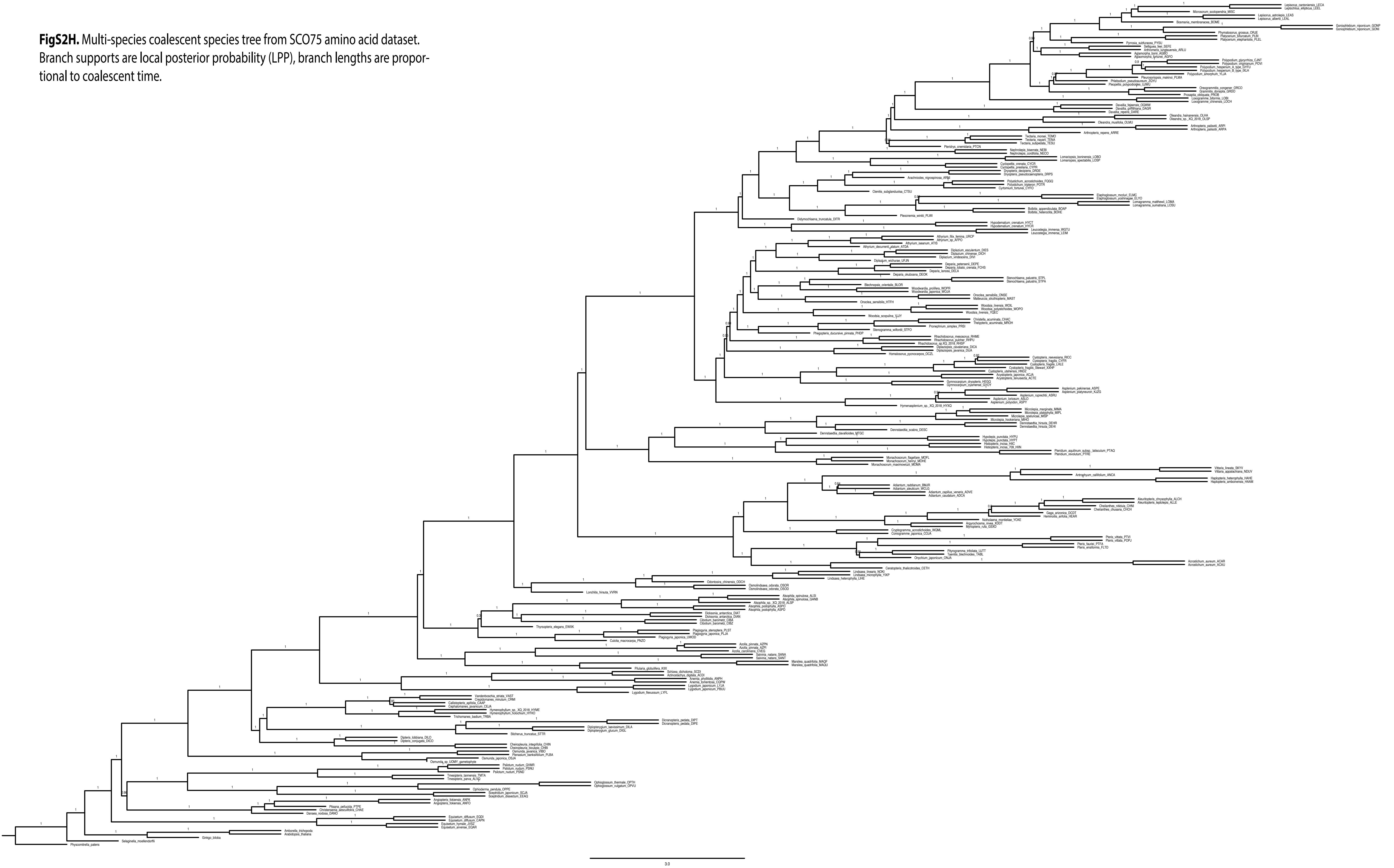

FigS2I. Maximum likelihood species tree from concatenated SCO60 nucleotide dataset. Branch supports are given as ultra-fast bootstrap support, branch lengths are proportional to number of substitutions per site.

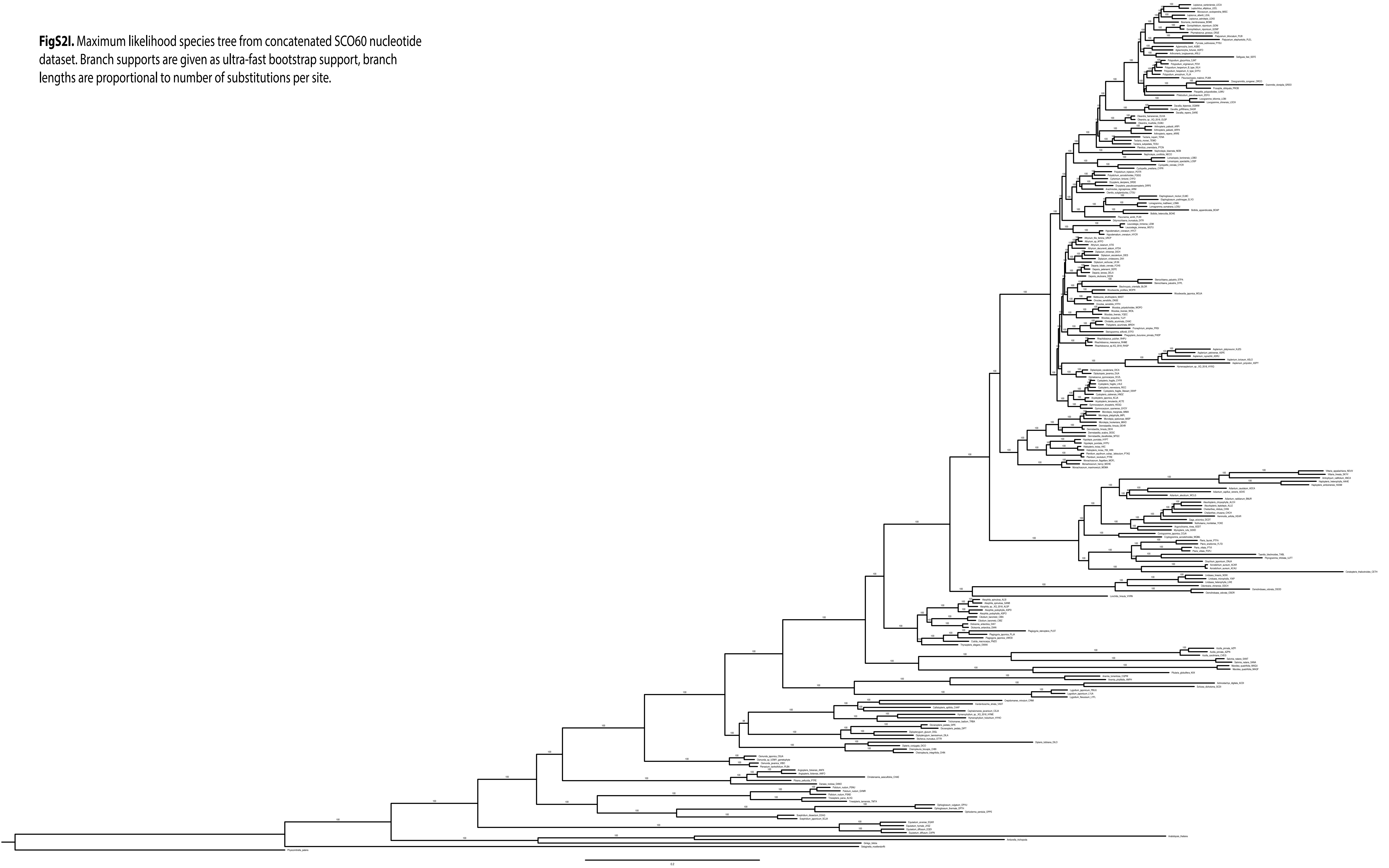

FigS2J. Maximum likelihood species tree form concatenated SCO60 amino acid dataset. Branch supports are given as ultra-fast bootstrap support, branch lengths are proportional to number of substitutions per site.

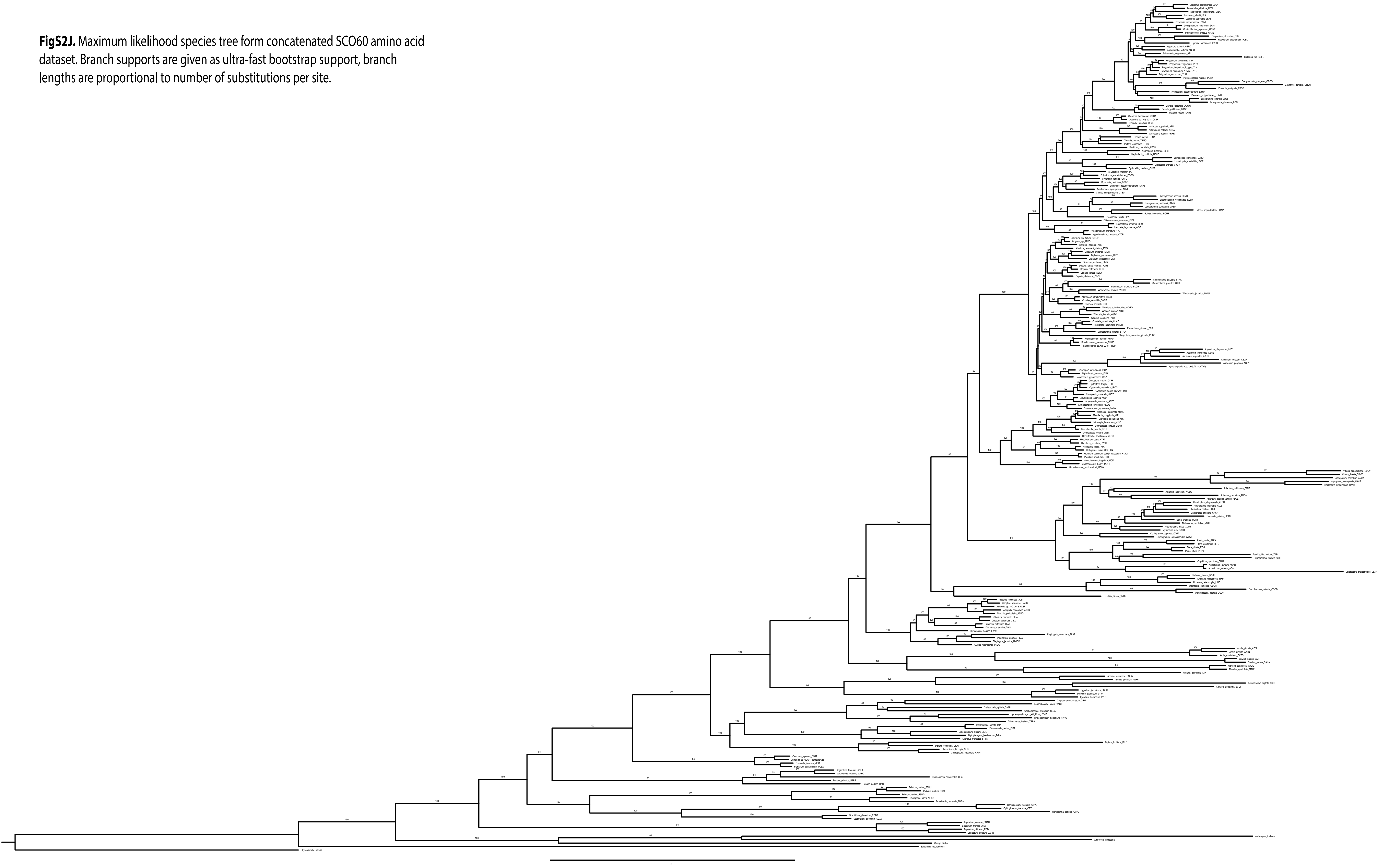

FigS2K. Multi-species coalescent species tree from SCO60 nucleotide dataset. Branch supports are local posterior probability (LPP), branch lengths are proportional to coalescent time.

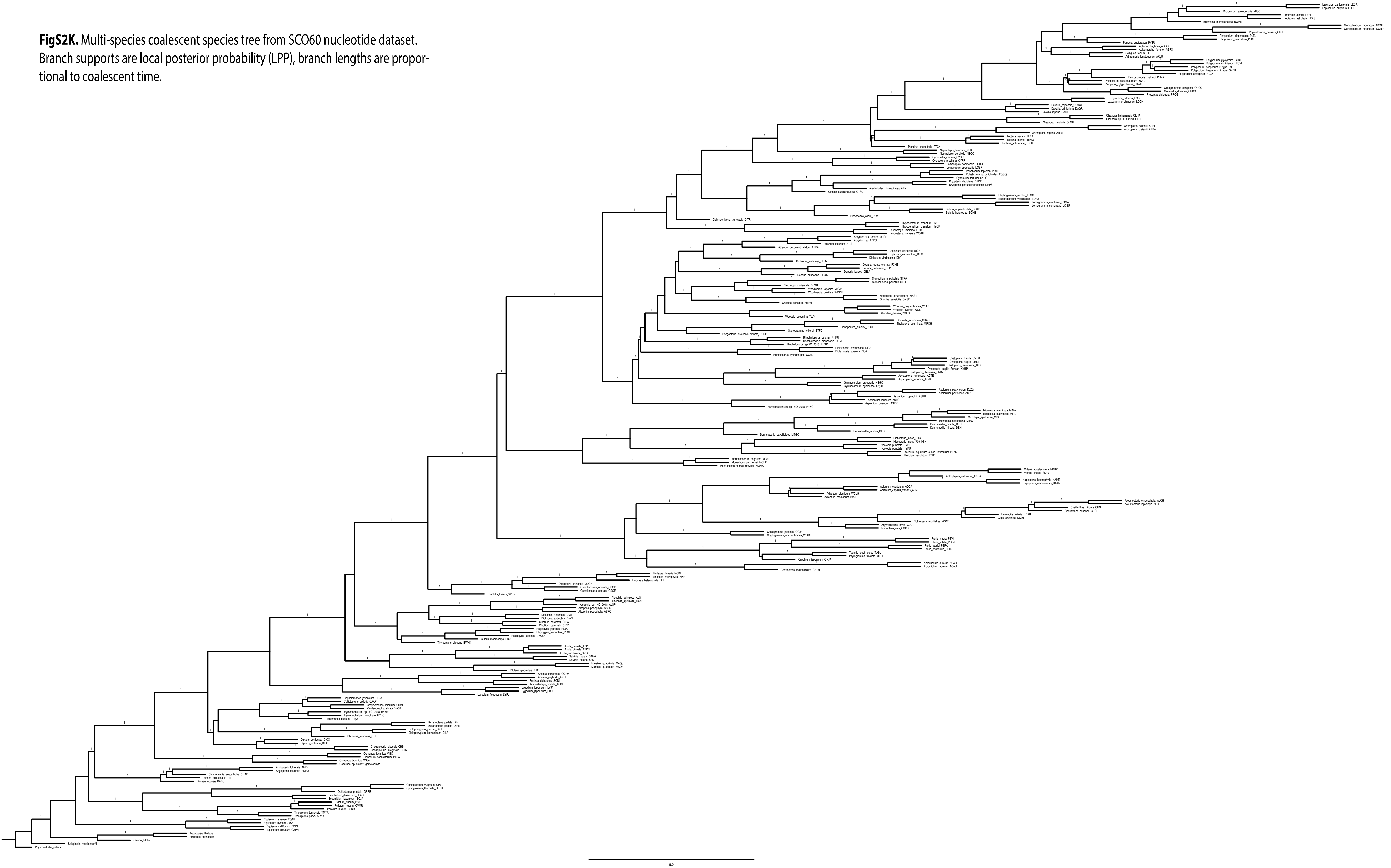

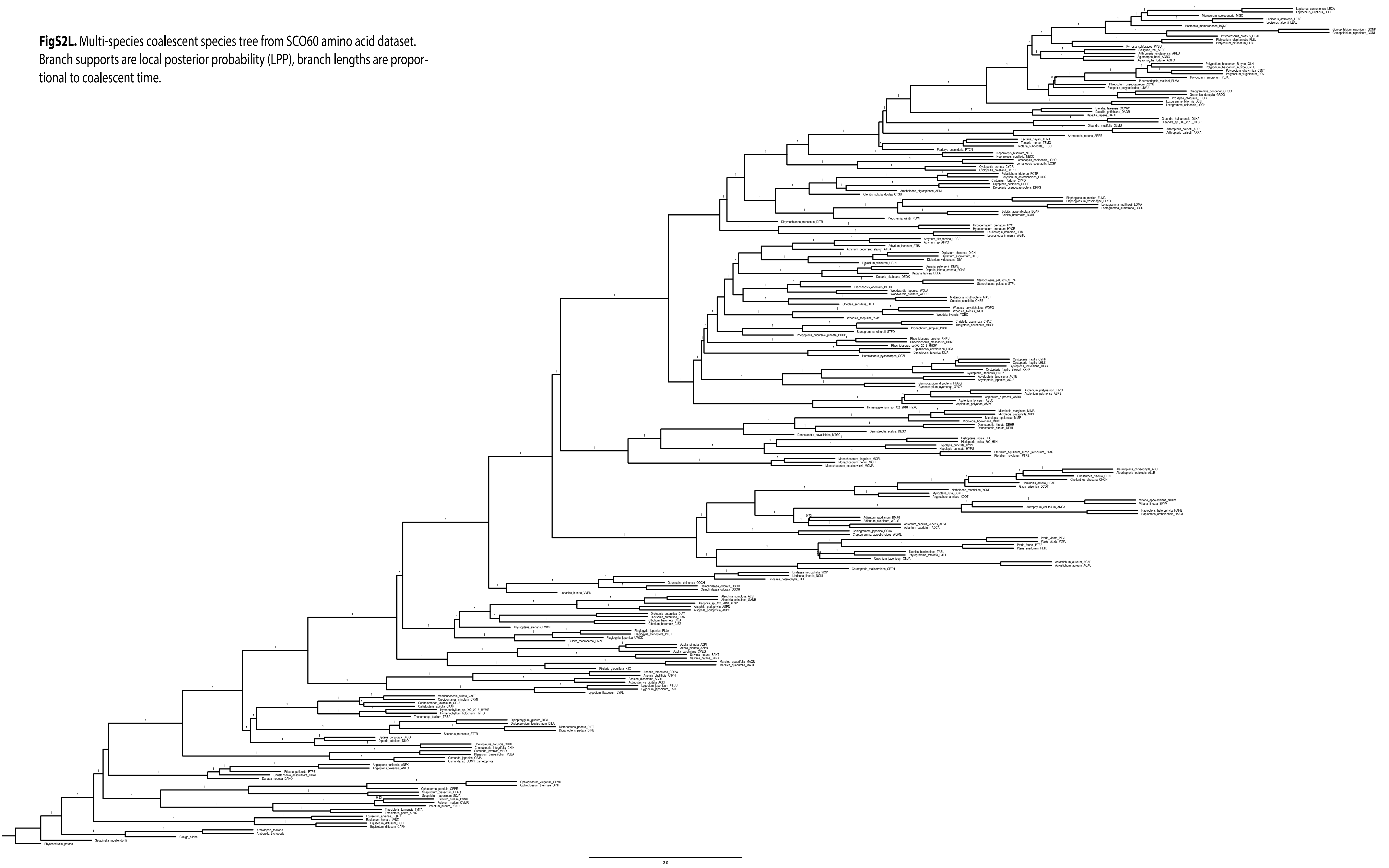

FigS2L. Multi-species coalescent species tree from SCO60 amino acid dataset. Branch supports are local posterior probability (LPP), branch lengths are proportional to coalescent time.

FigS2M. Multi-species coalescent species tree from MCO nucleotide dataset. Branch supports are local posterior probability (LPP), branch lengths are proportional to coalescent time.

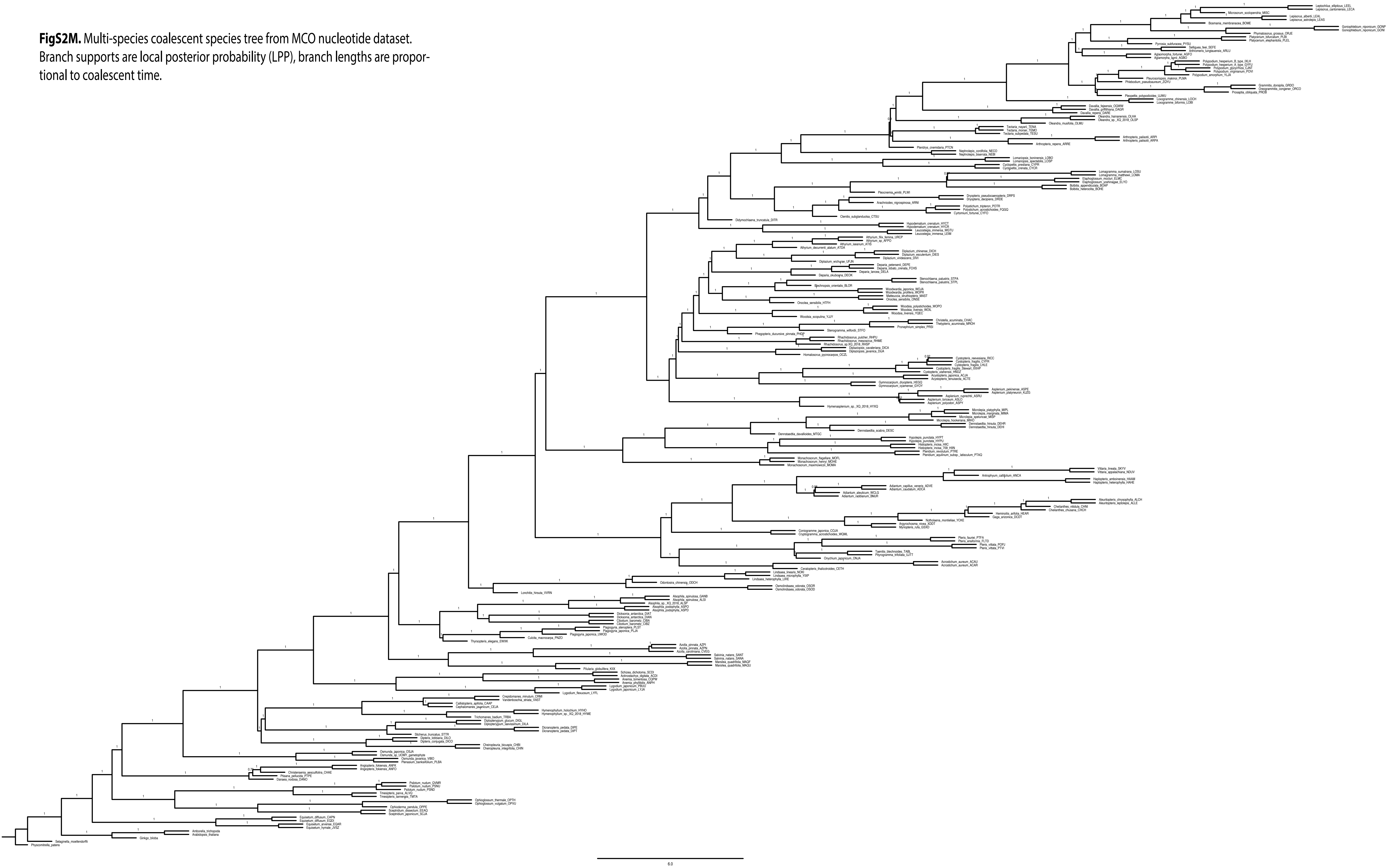

FigS2N. Multi-species coalescent species tree from MCO amino acid dataset. Branch supports are local posterior probability (LPP), branch lengths are proportional to coalescent time.

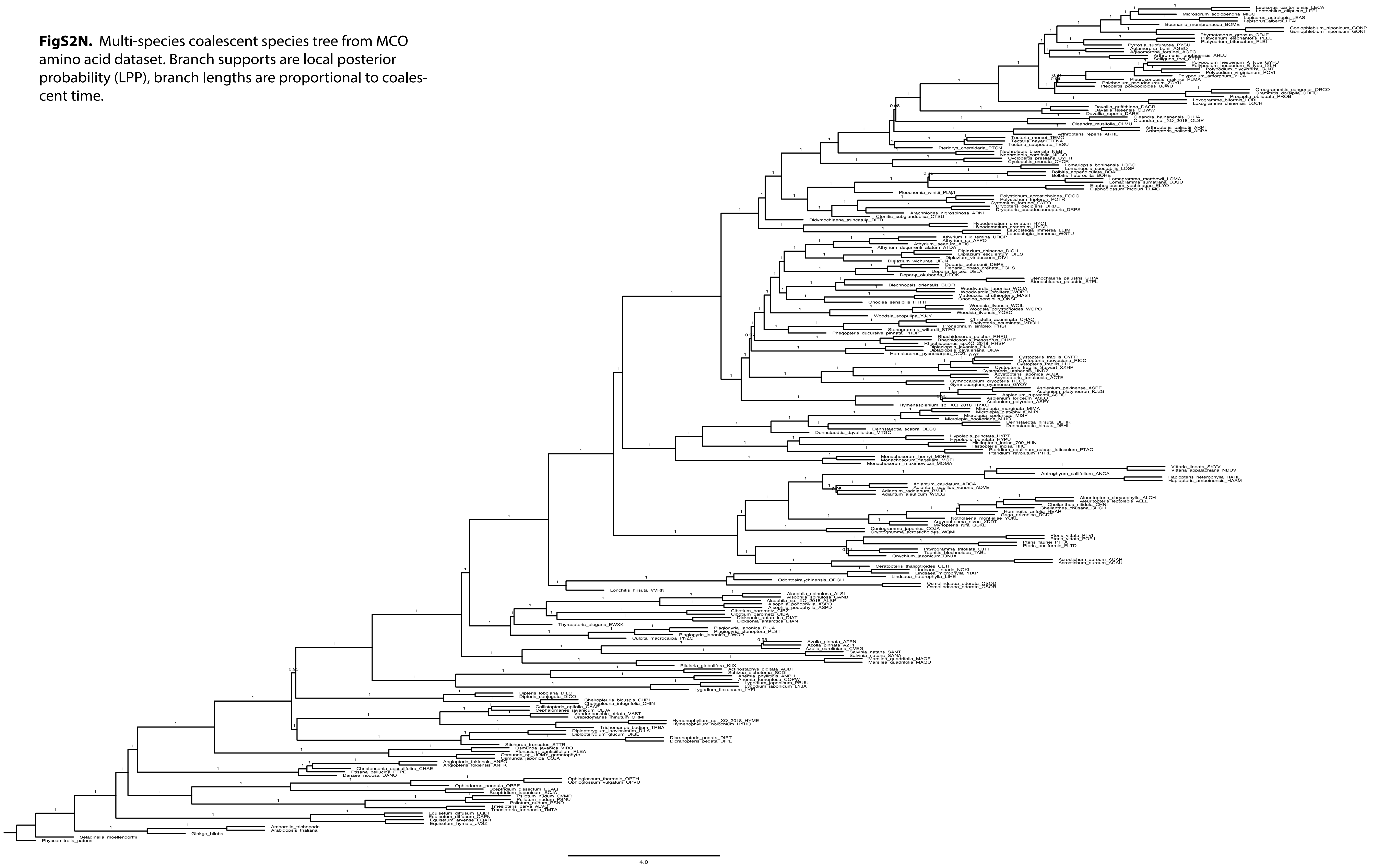

FigS20. Multi-species coalescent species tree from SCO60 nucleotide dataset with each locus run with the linked GHOST model. Branch supports are local posterior probability (LPP), branch lengths are proportional to coalescent time.

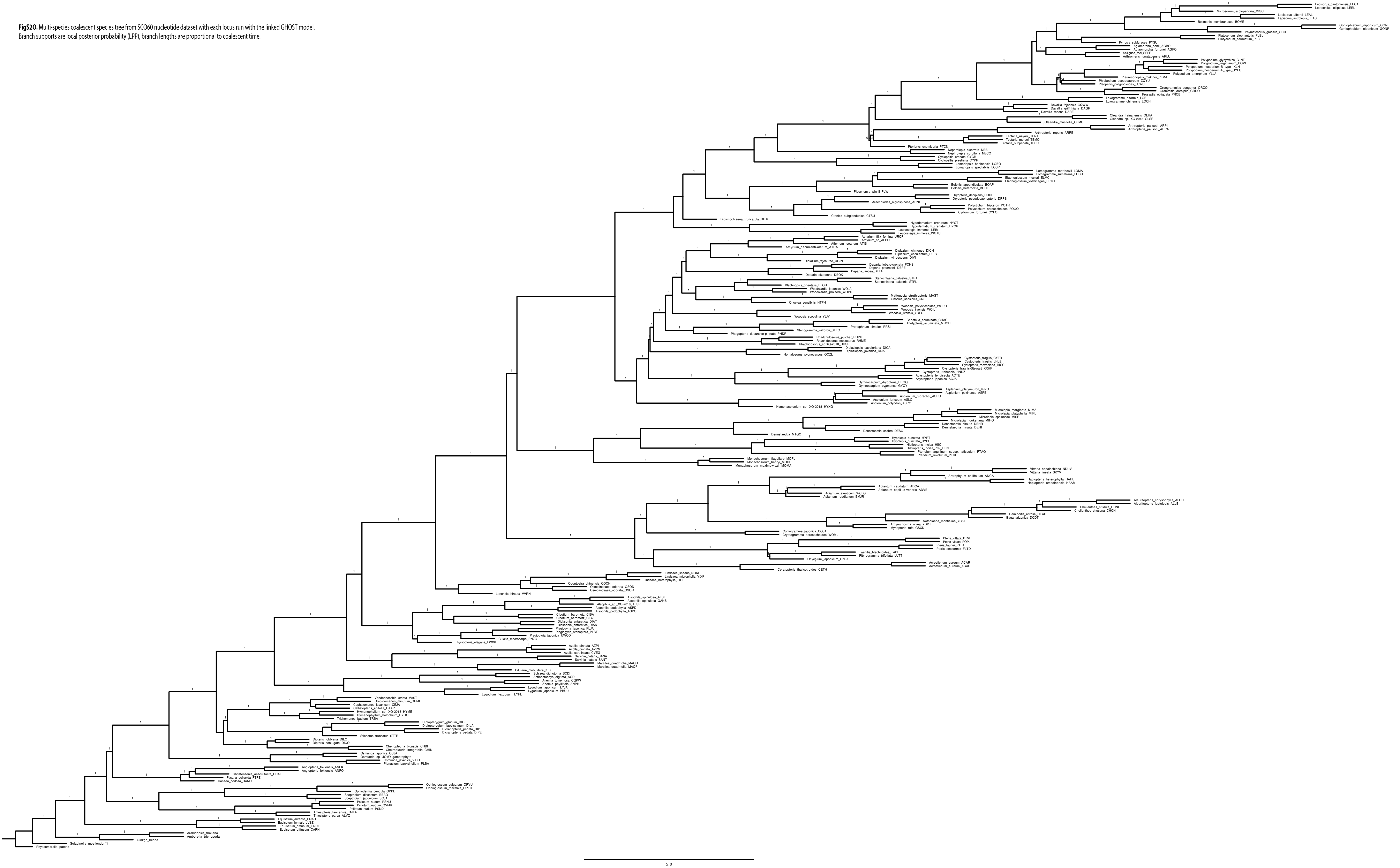

FigS2P. Multi-species coalescent species tree from SCO60 nucleotide dataset with each locus run with the unlinked GHOST model. Branch supports are local posterior probability (LPP), branch lengths are proportional to coalescent time.

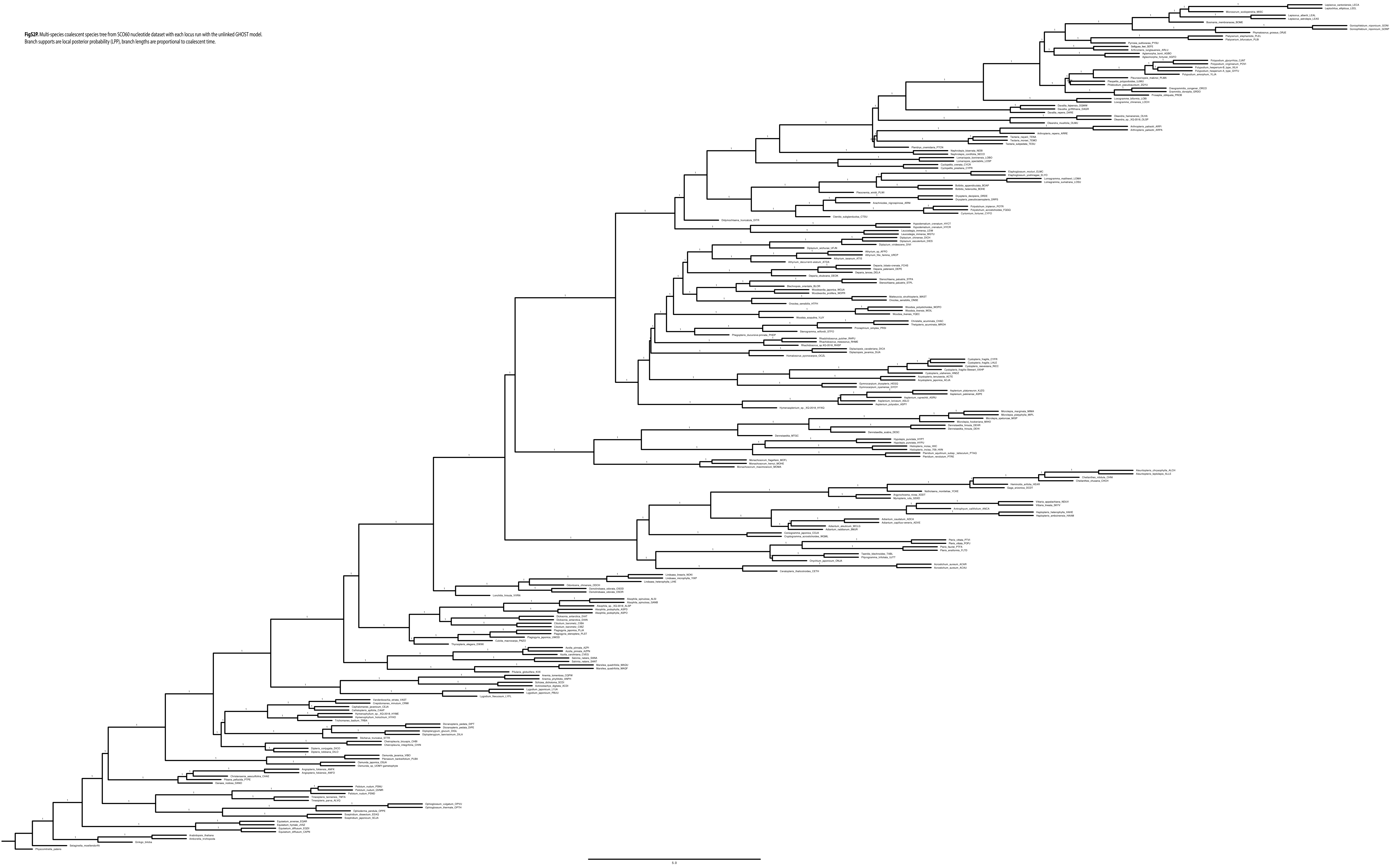

FigS2Q. Multi-species coalescent species tree from first and second codon positions of each locus in the SCO60 nucleotide dataset. Branch supports are local posterior probability (LPP), branch lengths are proportional to coalescent time.

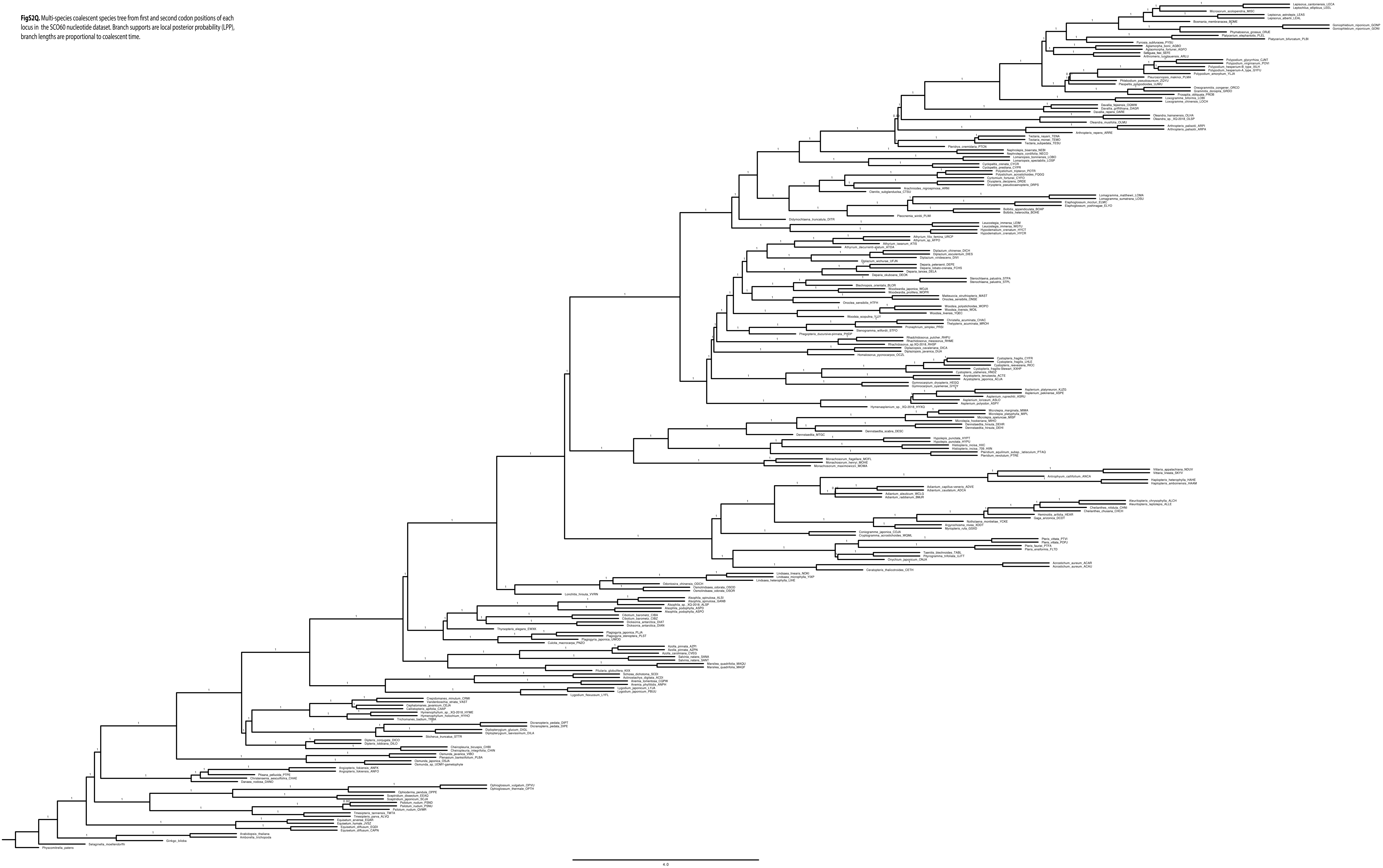

FigS2R. Multi-species coalescent species tree from the third codon position of each locus in the SCO60 nucleotide dataset. Branch supports are local posterior probability (LPP), branch lengths are proportional to coalescent time.

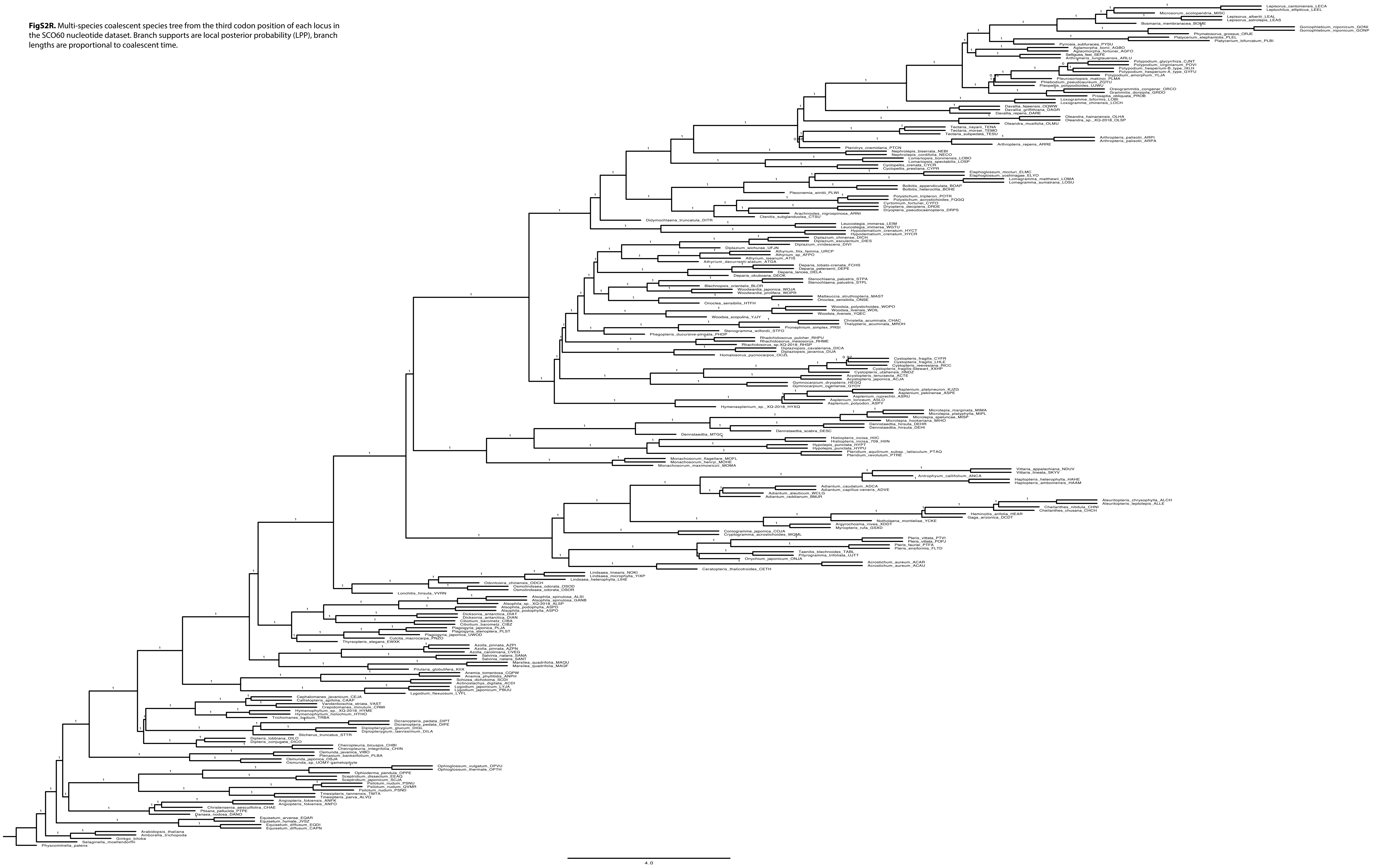

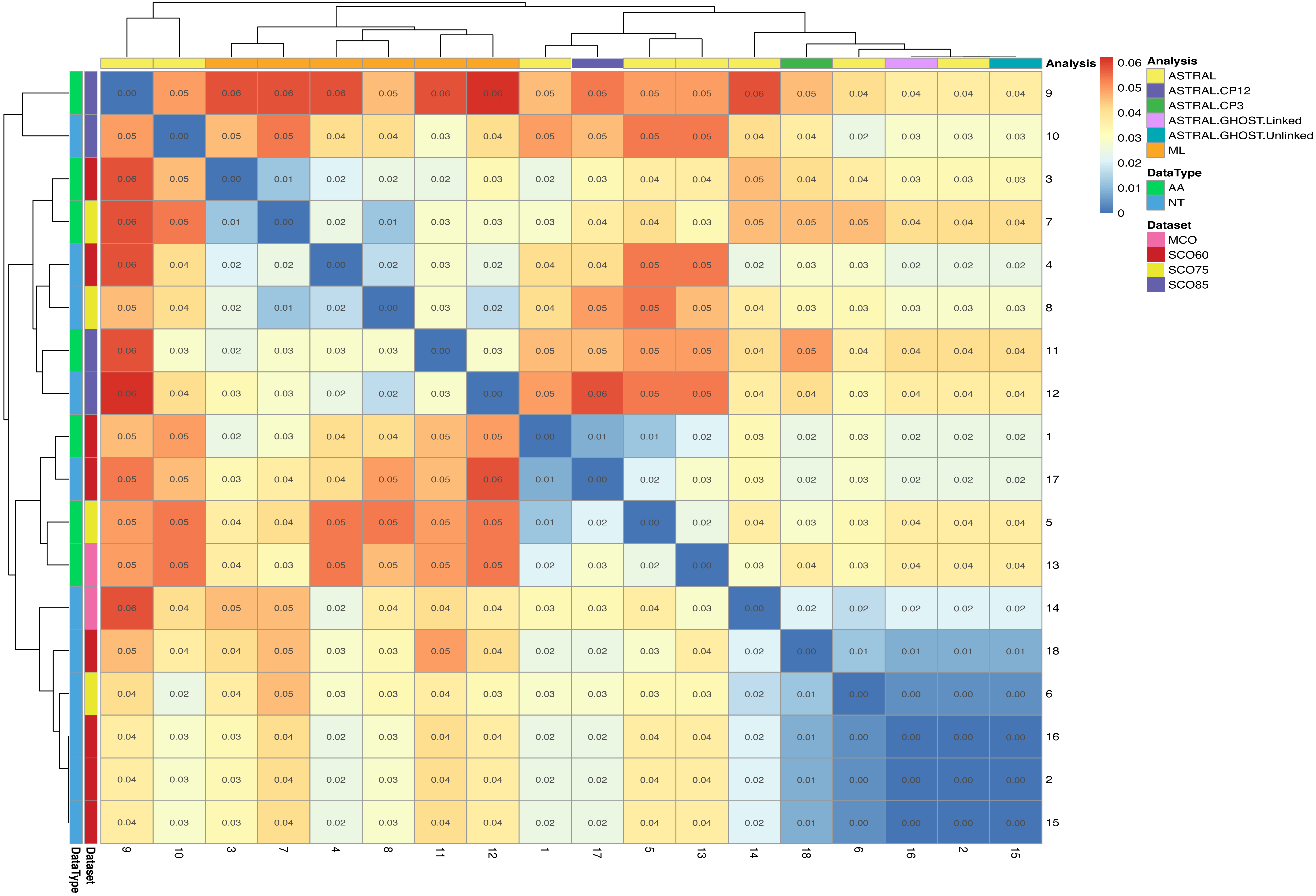

Supplemental Figure 3. Heatmap of generalized Robinson-Foulds distances between tree topologies from different analyses.

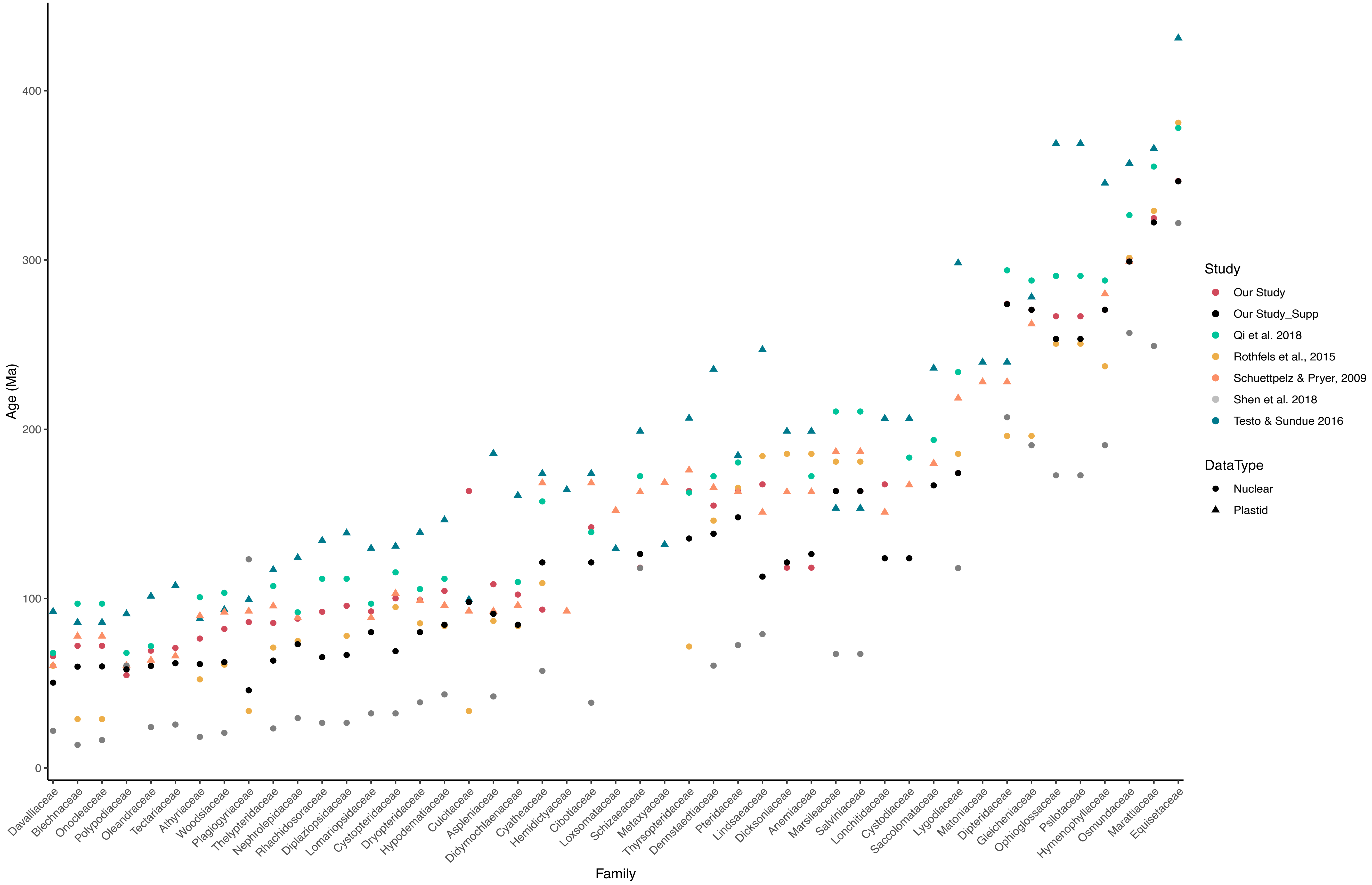

Supplemental Figure 4. Stem ages of fern families from Schuettpelz and Pryer (2009), Rothfels et al. (2015), Testo and Sundue (2016), Shen et al. (2018), Qi et al. (2018), and this study (main analysis wtih 15 fossil calibrations in red; supplemental analysis with 17 fossil calibrations in black).

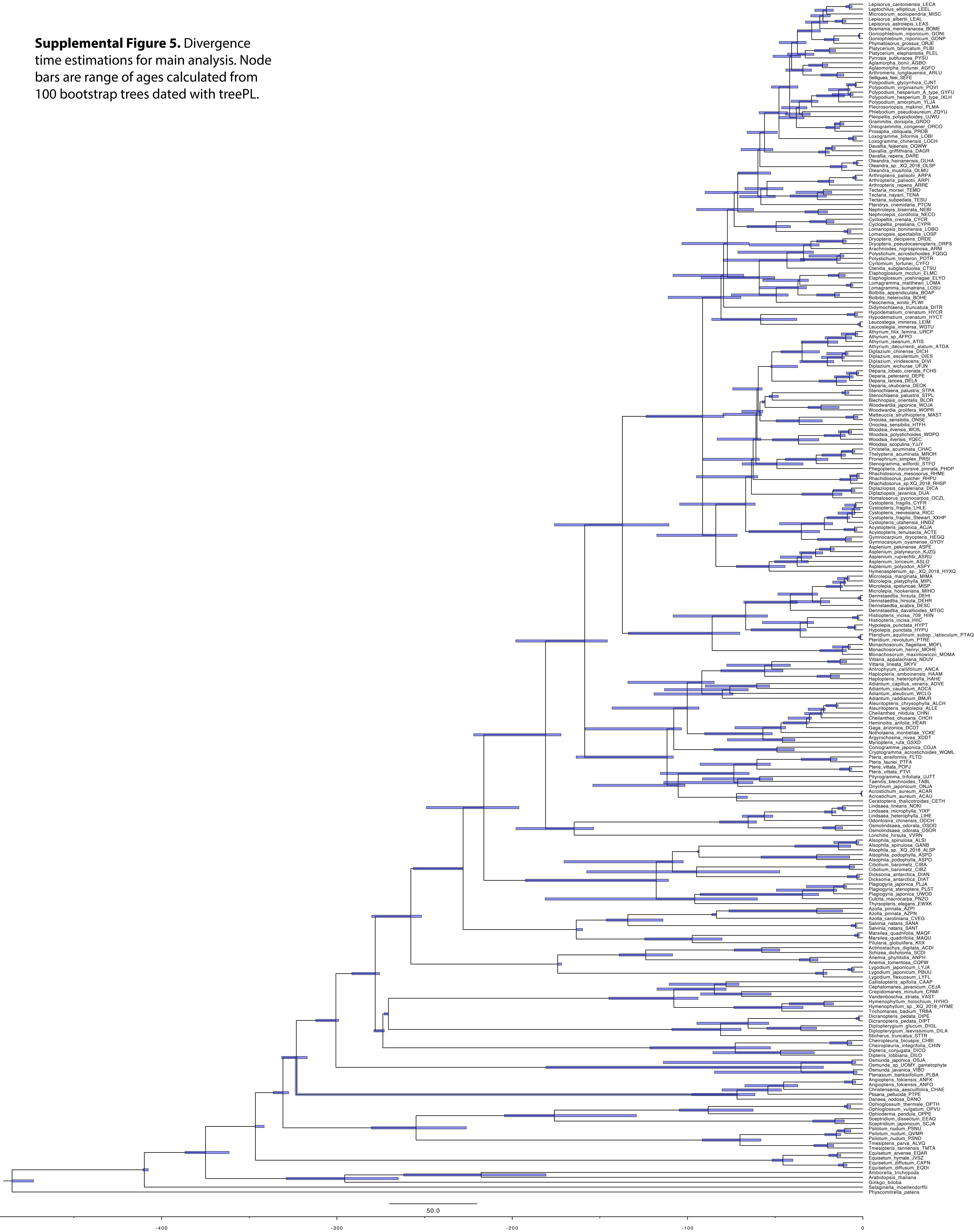

Supplemental Figure 5. Divergence time estimations for main analysis. Node bars are range of ages calculated from 100 bootstrap trees dated with treePL.

Supplemental Figure 6. Divergence time estimations for supplemental analysis. Node bars are range of ages calculated from 100 bootstrap trees dated with treePL.

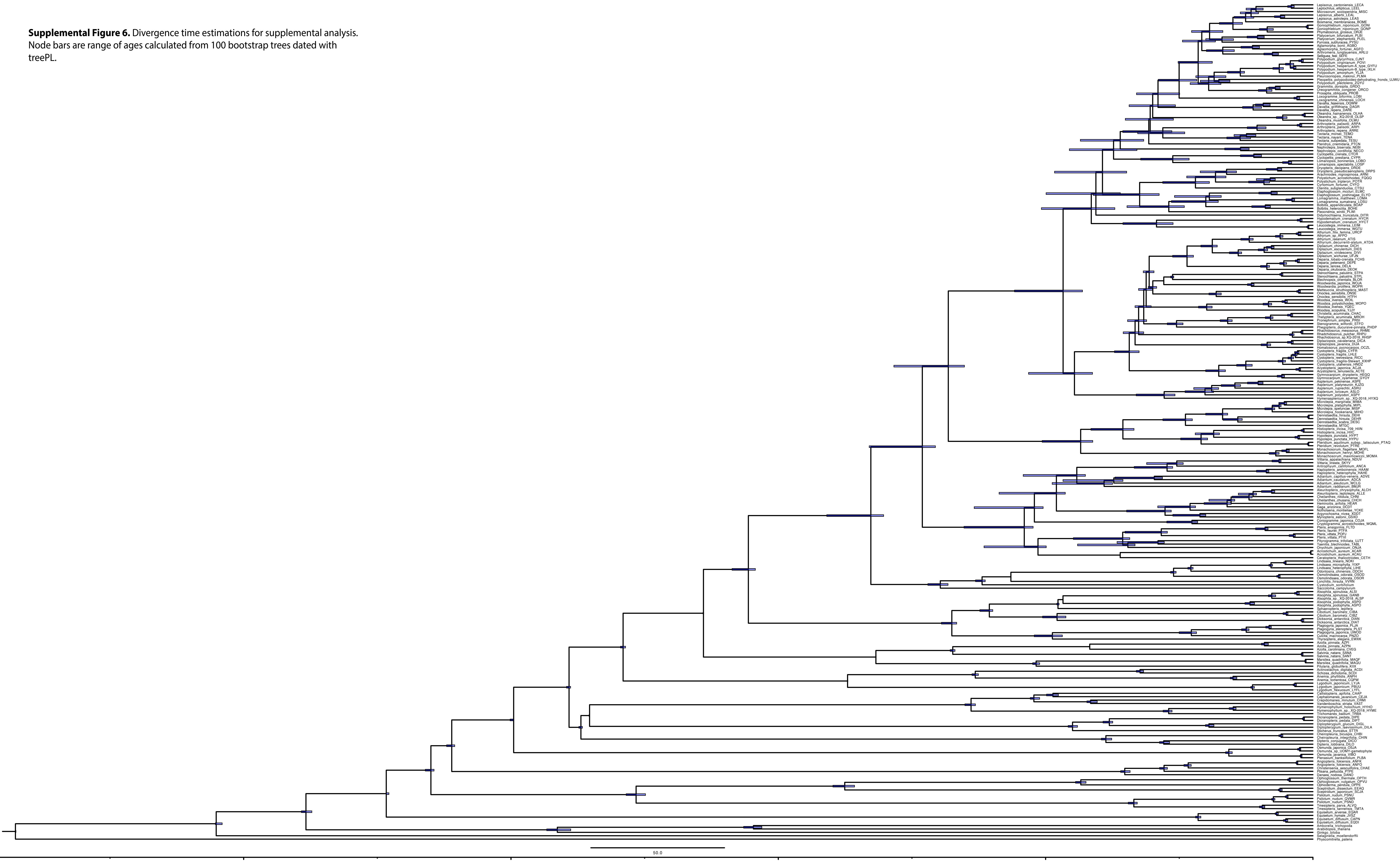

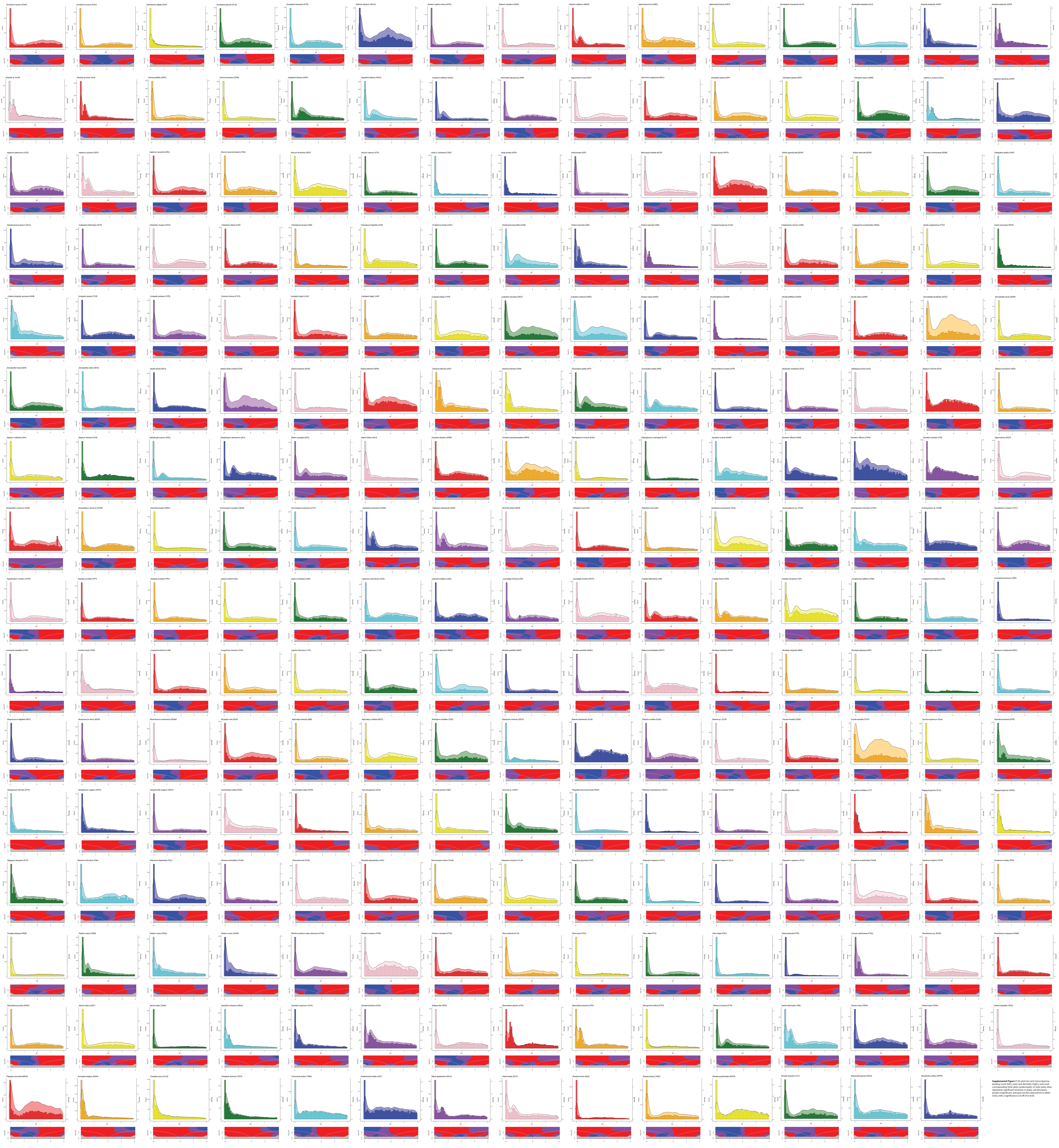

Supplemental Figure 7. KS plots for each transcriptome, paralog count (left y-axis) and densities (right y-axis) and corresponding SiZer plots (underneath). In SiZer plots, blue represents significant increases in slope, red decreases, purple insignificant, and grey too few data points to determine, with a significance cut-off of α=0.05.

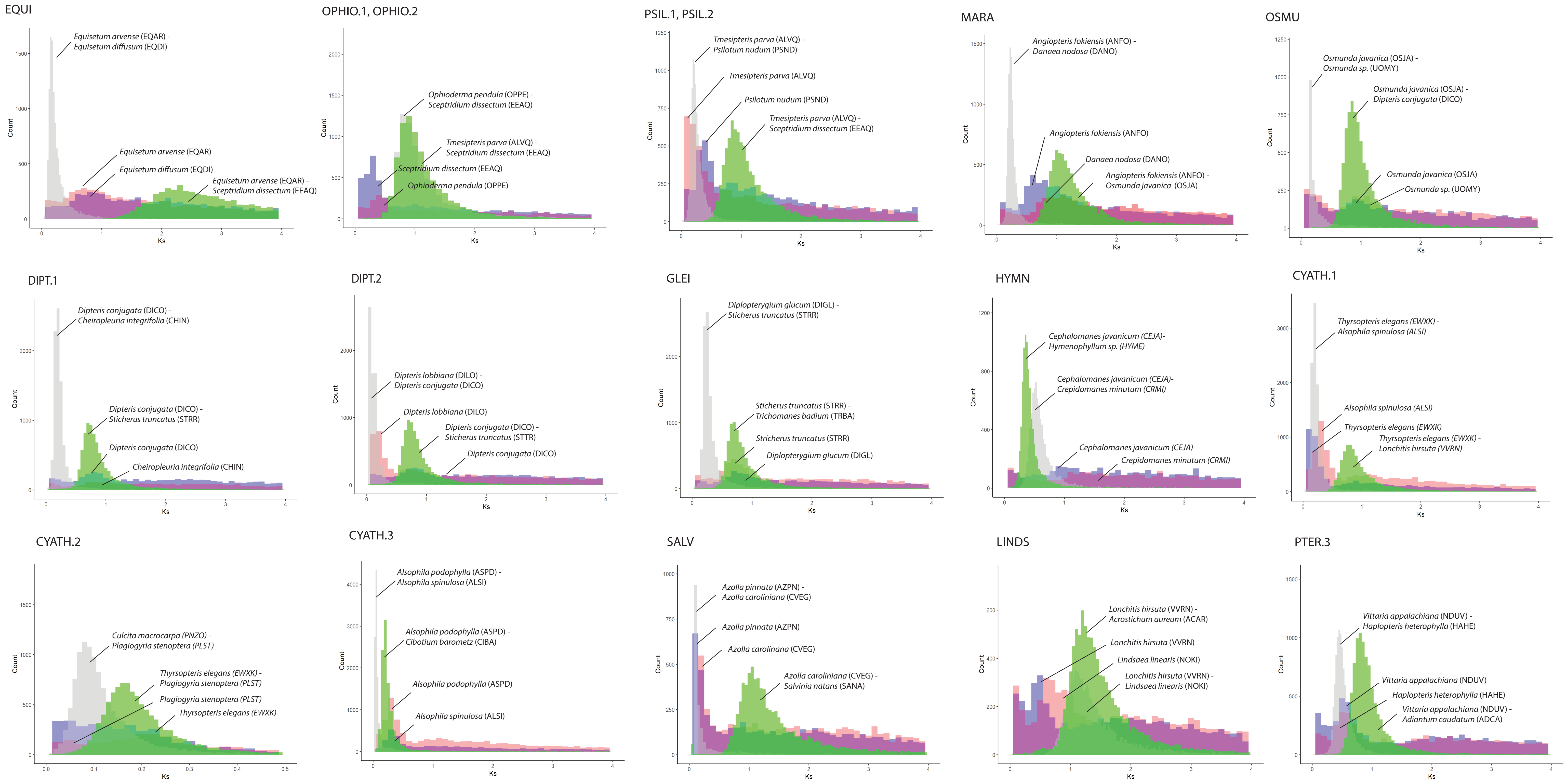

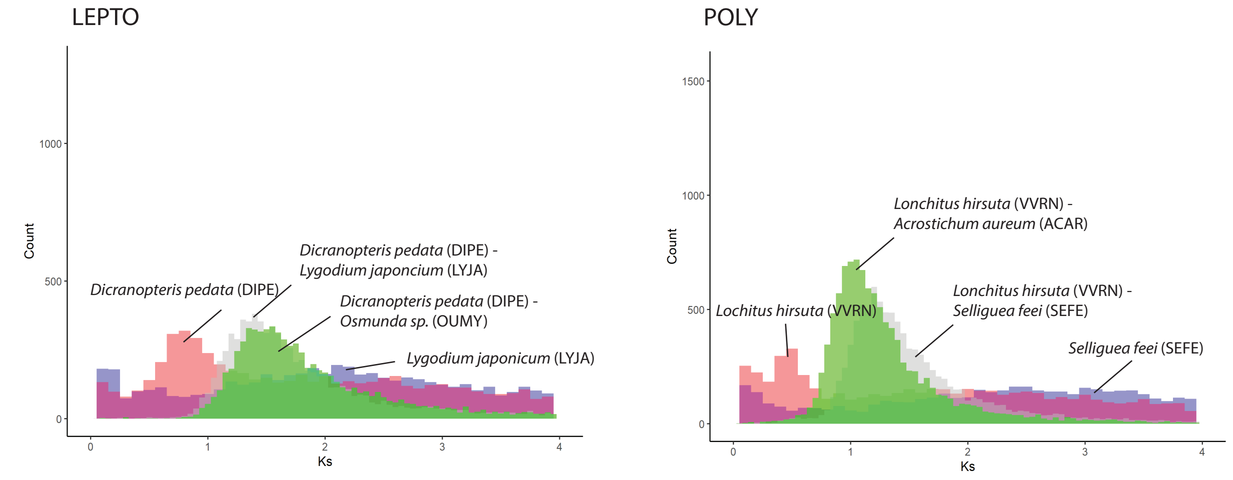

Supplemental Figure 8. Uncorrected synonymous divergence (Ks) between one-to-one orthologs of selected taxa for each large-scale event. Ks plots are also shown for individual taxa invovled in the event, either in red or blue. Older ortholog divergences (those with nodes deeper in the phylogeny) are shown in green, younger ortholog divergences (those with nodes shallower in the phylogeny) are shown in gray. Note that the relative timing of the inferred events is ambiguous. Furthermore, differences in rates of molecular evolution, may make older divergences appear to have occured more recently than younger divergences.

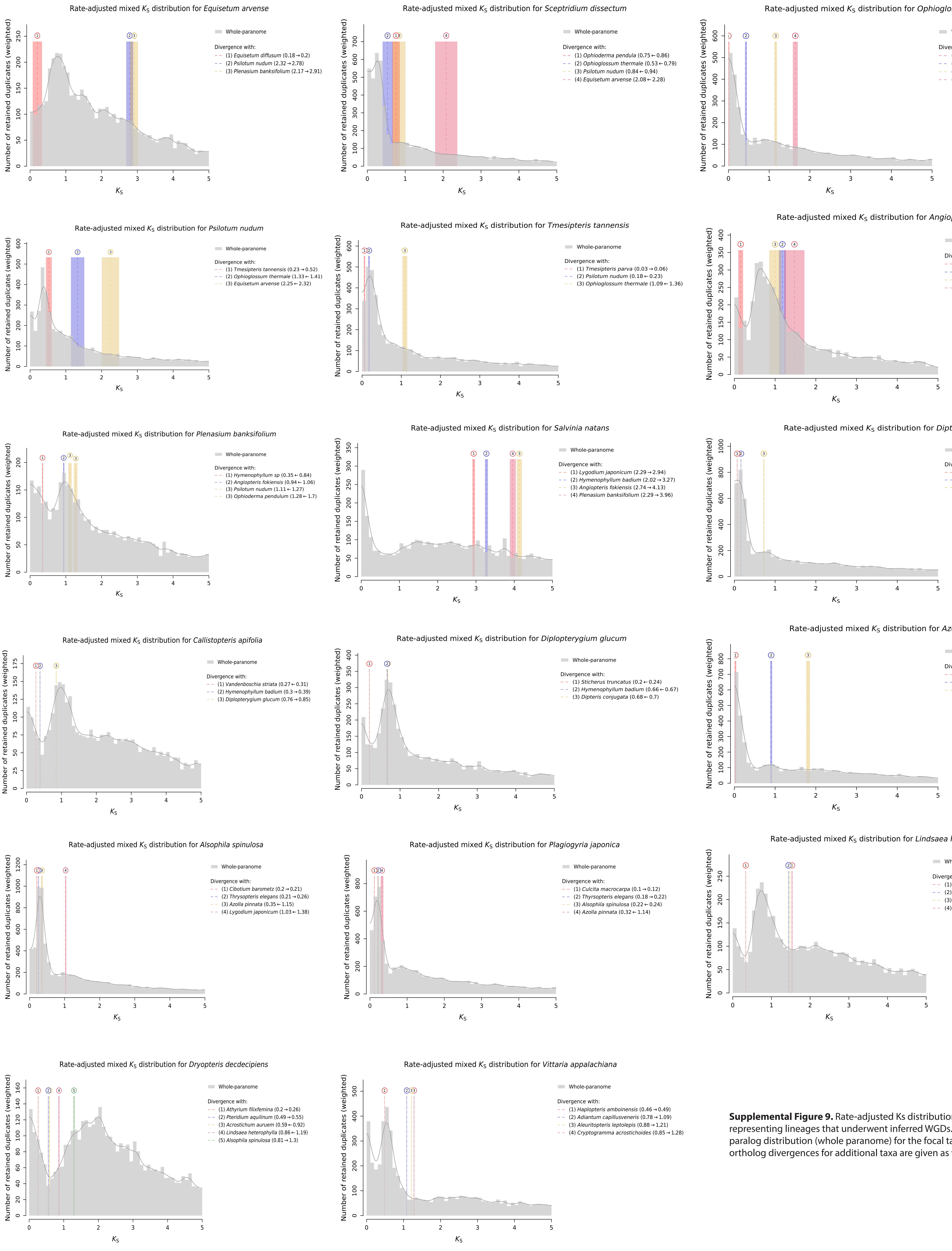

Supplemental Figure 9. Rate-adjusted Ks distributions for selected taxa representing lineages that underwent inferred WGDs. Gray distribution is the paralog distribution (whole paranome) for the focal taxon. Rate-corrected ortholog divergences for additional taxa are given as vertical colored lines.

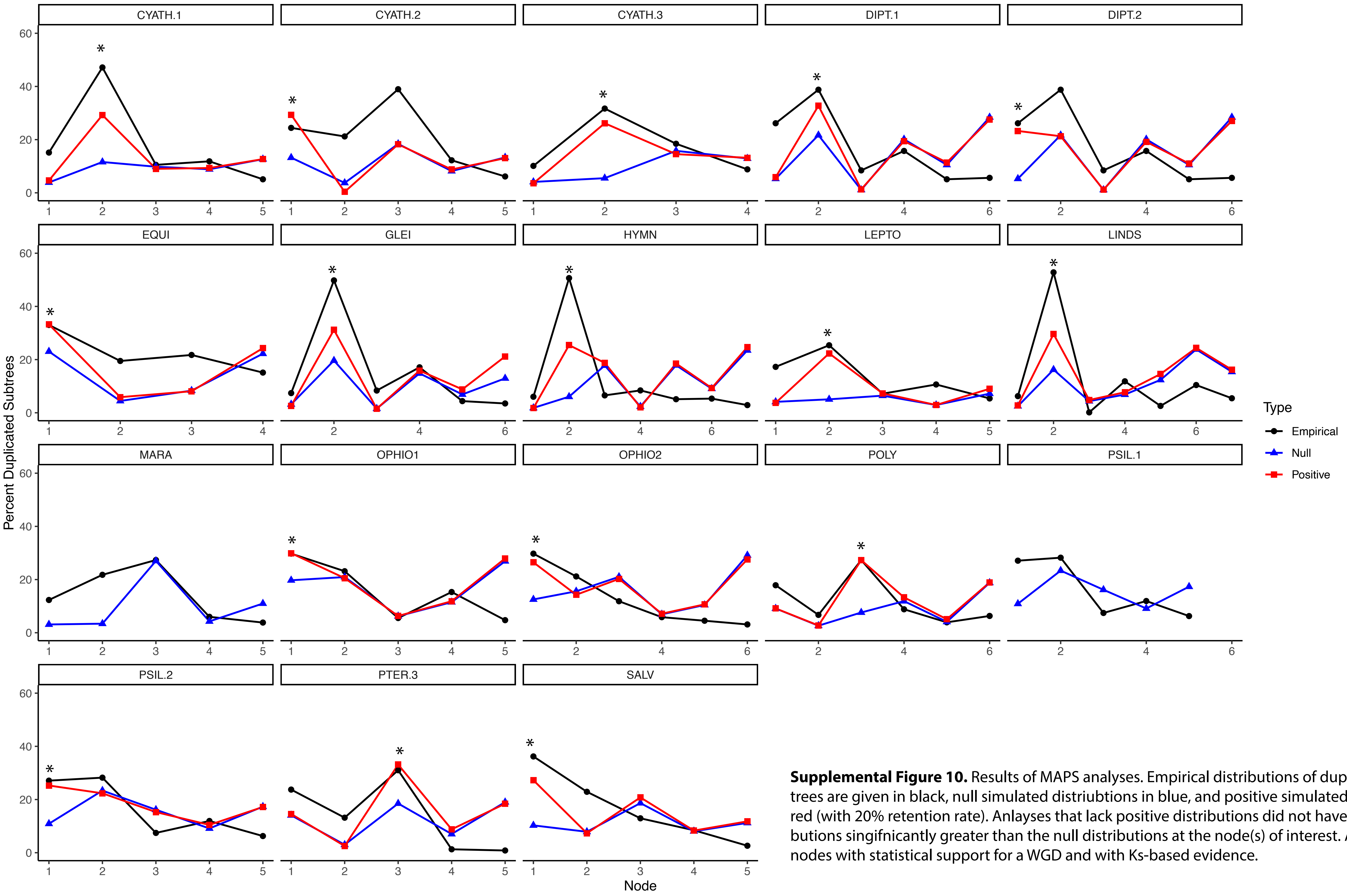

Supplemental Figure 10. Results of MAPS analyses. Empirical distributions of duplicated gene trees are given in black, null simulated distriubtions in blue, and positive simulated distributions in red (with 20% retention rate). Anlayses that lack positive distributions did not have empirical distributions singifnicantly greater than the null distributions at the node(s) of interest. Asterisks denote nodes with statistical support for a WGD and with Ks-based evidence.

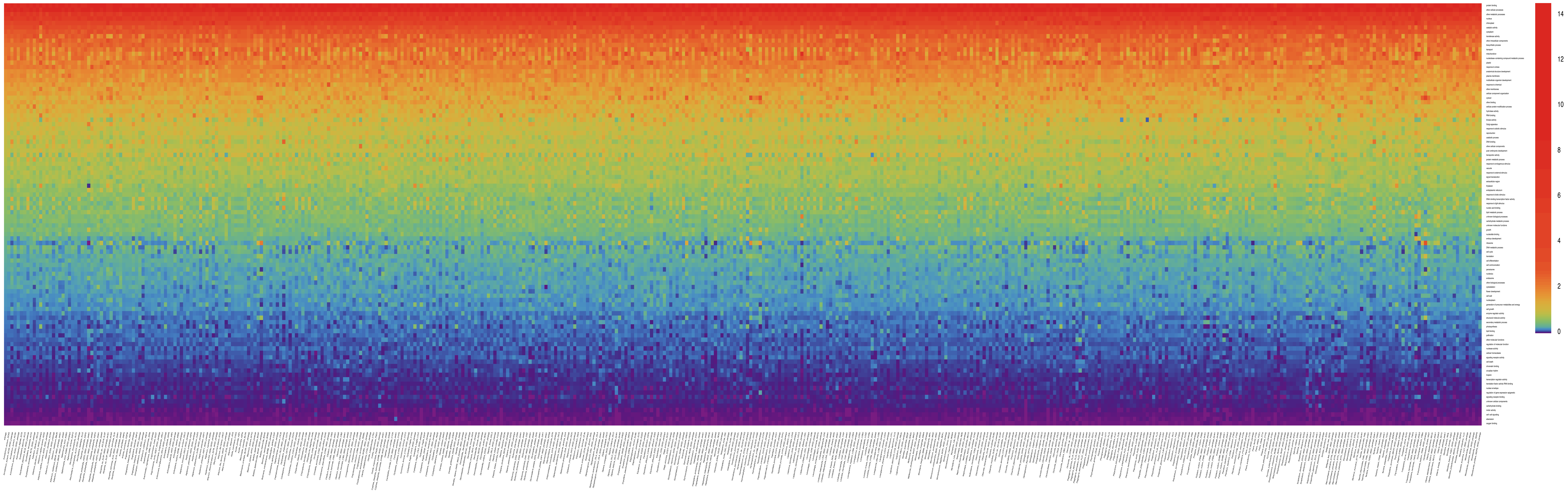

Supplemental Figure 11. Heatmap of the proportion of transcriptomes and associated paralogs (retained duplicates) associated with GO Slim categories as annotated with the GOGetter pipeline. GO Slim cateogories are from the annotated *Arabidopsis thaliana* TAIR21 genome. Colors of the heamap cells represent the proportion of the gene set (either full transcriptome or paralogs) corresponding to each GO Slim category, with warmer colors representing higher proportions.

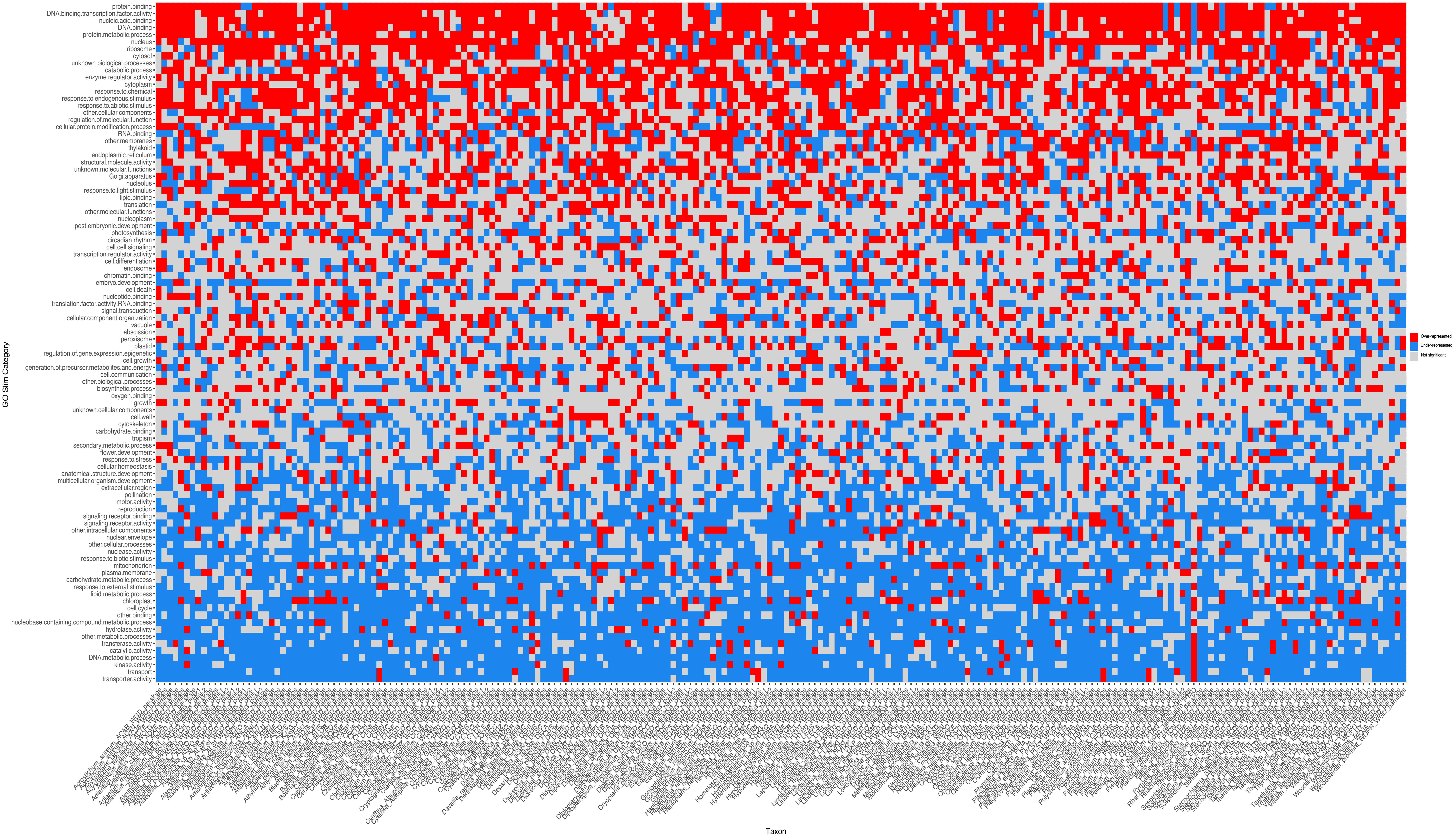

Supplemental Figure 12. Heatmap of GO Slim categories that are over-represented (red), under-represented (blue), or not significant (gray) in paralogs that correspond to whole genome duplication (WGD) events compared to full transcriptomes for each taxon with evidence of WGD in their Ks plots. Significance was determined using residuals from chi-squared tests following Barker et al. (2008).
